# Energetic insights into two electron transfer pathways in light-driven energy-converting enzymes†
†Electronic supplementary information (ESI) available. See DOI: 10.1039/c8sc00424b


**DOI:** 10.1039/c8sc00424b

**Published:** 2018-03-28

**Authors:** Keisuke Kawashima, Hiroshi Ishikita

**Affiliations:** a Department of Applied Chemistry , The University of Tokyo , 7-3-1 Hongo, Bunkyo-ku , Tokyo 113-8654 , Japan . Email: hiro@appchem.t.u-tokyo.ac.jp; b Research Center for Advanced Science and Technology , The University of Tokyo , 4-6-1 Komaba, Meguro-ku , Tokyo 153-8904 , Japan . Fax: +81-3-5452-5083 ; Tel: +81-3-5452-5056

## Abstract

We report redox potentials (*E*_m_) for one-electron reduction for all chlorophylls in the two electron-transfer branches of water-oxidizing enzyme photosystem II (PSII), photosystem I (PSI), and purple bacterial photosynthetic reaction centers (PbRC). In PSI, *E*_m_ values for the accessory chlorophylls were similar in both electron-transfer branches. In PbRC, the corresponding *E*_m_ value was 170 mV less negative in the active L-branch (B_L_) than in the inactive M-branch (B_M_), favoring B_L_˙^–^ formation. This contrasted with the corresponding chlorophylls, Chl_D1_ and Chl_D2_, in PSII, where *E*_m_(Chl_D1_) was 120 mV more negative than *E*_m_(Chl_D2_), implying that to rationalize electron transfer in the D1-branch, Chl_D1_ would need to serve as the primary electron donor. Residues that contributed to *E*_m_(Chl_D1_) < *E*_m_(Chl_D2_) simultaneously played a key role in (i) releasing protons from the substrate water molecules and (ii) contributing to the larger cationic population on the chlorophyll closest to the Mn_4_CaO_5_ cluster (P_D1_), favoring electron transfer from water molecules. These features seem to be the nature of PSII, which needs to possess the proton-exit pathway to use a protonated electron source—water molecules.

## Abstract 2

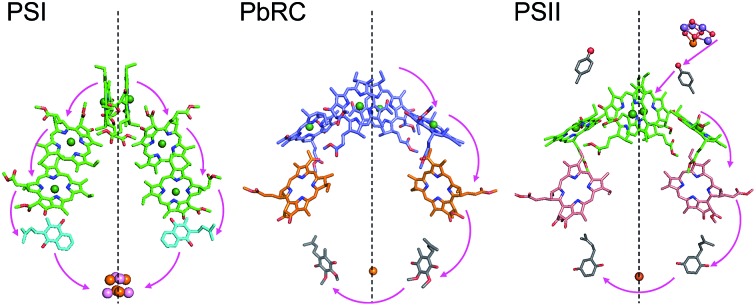
We report *E*_m_ values of (bacterio-)chlorophylls for one-electron reduction in both electron-transfer branches of PbRC, PSI, and PSII.

## 

The crystal structures of photosystem II (PSII), photosystem I (PSI) and purple bacterial photosynthetic reaction centers from *Rhodobacter sphaeroides* (PbRC) show a pseudo-twofold axis of symmetry, forming the following heterodimeric protein subunit pairs: D1/D2 in PSII, PsaA/PsaB in PSI, and L/M in PbRC.^1^–^6^ In PbRC, electron-transfer branches (L- and M-branches) proceed from a pair of bacteriochlorophyll *a* (BChl*a*) (P_L_ and P_M_) *via* accessory BChl*a* (B_L_ and B_M_), bacteriopheophytin *a* (BPheo*a*) (H_L_ and H_M_), and ubiquinone (Q_A_ and Q_B_). In PSII, the corresponding cofactors are the pair of chlorophyll *a* (Chl*a*) (P_D1_ and P_D2_), accessory Chl*a* (Chl_D1_ and Chl_D2_), pheophytin *a* (Pheo*a*) (Pheo_D1_ and Pheo_D2_), and plastoquinone (Q_A_ and Q_B_) of D1- and D2-branches, and in PSI, the pair of Chl*a* and the 13^2^ epimer^7^ (P_A_ and P_B_), accessory Chl*a* (A_–1A_ and A_–1B_), acceptor Chl*a* (A_0A_ and A_0B_), and phylloquinone (A_1A_ and A_1B_) of A- and B-branches (Fig. 1). In PSI (*i.e.*, a type-I reaction center), electron transfer occurs in both A- and B-branches,^8^ whereas in PbRC and PSII (*i.e.*, type-II reaction centers) electron transfer predominantly occurs along L- and D1-branches, respectively. In PbRC and PSII, excitation of BChl*a* and Chl*a* leads to charge separation on the L- and D1-branches and formation of the cationic [P_L_/P_M_]˙^+^ and [P_D1_/P_D2_]˙^+^ states, respectively (*e.g.*,^9^). Regardless of the structural similarities between the two reaction centers,^1^ many features are different.^9^ The [P_L_/P_M_]˙^+^ state has a redox potential (*E*_m_) of 500 mV for one-electron oxidation^10^ and accepts an electron from an outer protein subunit, cytochrome *c*_2_ (or tetraheme cytochrome in PbRC from *Blastochloris viridis*). The [P_D1_/P_D2_]˙^+^ state has a high *E*_m_ (>1100 mV)^11^–^13^ and ultimately abstracts electrons from the substrate water molecules at the catalytic Mn_4_CaO_5_ moiety in D1 *via* redox-active D1-Tyr161 (TyrZ). Redox-active D2-Tyr160 (TyrD) exists at the symmetrical position in D2. Basic D2-Arg180 and D2-His61 near TyrD on the D2 side contribute to the larger P_D1_˙^+^ population than P_D2_˙^+^ in [P_D1_/P_D2_]˙^+^,^13^*i.e.*, electrostatically pushing the cation onto P_D1_,^14^ thereby favoring electron transfer from the substrate water molecules in D1.^15^ Unlike PbRC, which only requires an electron transfer pathway, PSII also requires a proton transfer pathway from the substrate water molecules because the water molecules are protonated electron sources. In PSII, the release of protons (H^+^) has been observed in response to changes in the oxidation state (S_*n*_) of the oxygen-evolving complex, and it occurs with a typical stoichiometry of 1 : 0 : 1 : 2 for the S_0_ → S_1_ → S_2_ → S_3_ → S_0_ transitions, respectively.^16^ Proton transfer may proceed *via* different pathways depending on the S-state transitions.^17^–^19^ The nature of the proton-conducting O4-water chain,^20^ which is composed exclusively of water molecules, is consistent with and may explain the pH-independence of proton transfer in the S_0_ to S_1_ transition.^16^ On the other hand, the pH-dependent rate constant for the S_2_-to-S_3_ transition^16^ indicates the involvement of ionizable groups in the proton transfer pathway (*e.g.*, the pathway *via* D1-Asp61 21,22). Acquirement of the Mn_4_CaO_5_ cluster seems to induce a polar protein environment near the electron acceptor [P_D1_/P_D2_]˙^+^ and may alter the energetics of electron transfer along the D1-branch with respect to that along the L-branch in PbRC.

The free energy difference between cofactors (*e.g.*, electronically excited [P_L_/P_M_]* and [P_L_/P_M_]˙^+^H_L_˙^–^) was discussed experimentally (*e.g.*,^23^) and theoretically (*e.g.*,^24^). However, detailed *E*_m_ values of the cofactors for one-electron reduction in active L- and D1-branches as well as inactive M- and D2-branches have not yet been experimentally determined and are a matter of debate (*e.g.*,^25^). Although we reported calculated *E*_m_ values for one-electron oxidation in PSI, PbRC, and PSII,^13^,^26^ these *E*_m_ values are more associated with distributions of the cationic states over the (B)Chl*a* pairs (*e.g.*, [P_A_/P_B_]˙^+^ (ref. 27) and [P_D1_/P_D2_]˙^+^ (ref. 13)). Due to a lack of *E*_m_ values of the cofactors for one-electron reduction in PbRC, PSI, and PSII, it remains still unclear why electron transfer occurs in both A- and B-branches in PSI, whereas in PbRC and PSII electron transfer predominantly occurs along L- and D1-branches.

**Fig. 1:**
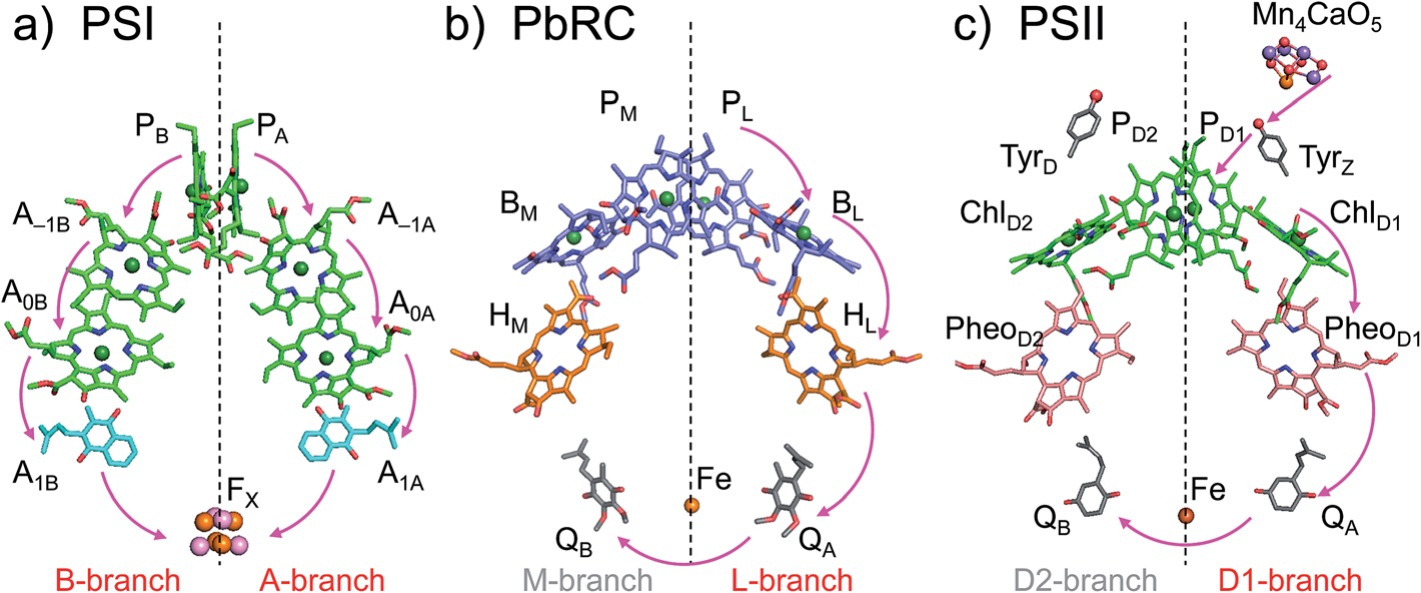
Electron transfer chains in photosynthetic reaction centers of (a) PSI (PDB code 1JB0), (b) PbRC (PDB code ; 3I4D), and (c) PSII (PDB code ; 3ARC). Pink arrows indicate electron transfer. Dotted lines indicate pseudo-*C*_2_ axes. Electron-transfer active branches are red labeled, whereas inactive branches are gray labeled.

Here, we present *E*_m_ values of Chl*a*, Pheo*a*, BChl*a*, and BPheo*a* for one-electron reduction in both electron-transfer branches of PbRC, PSI, and PSII; the *E*_m_ values were calculated using the crystal structures, solving the linear Poisson–Boltzmann equation, and considering the protonation states of all titratable sites in the entire proteins.

## Results

### *E*_m_ for accessory chlorophylls

**Fig. 2:**
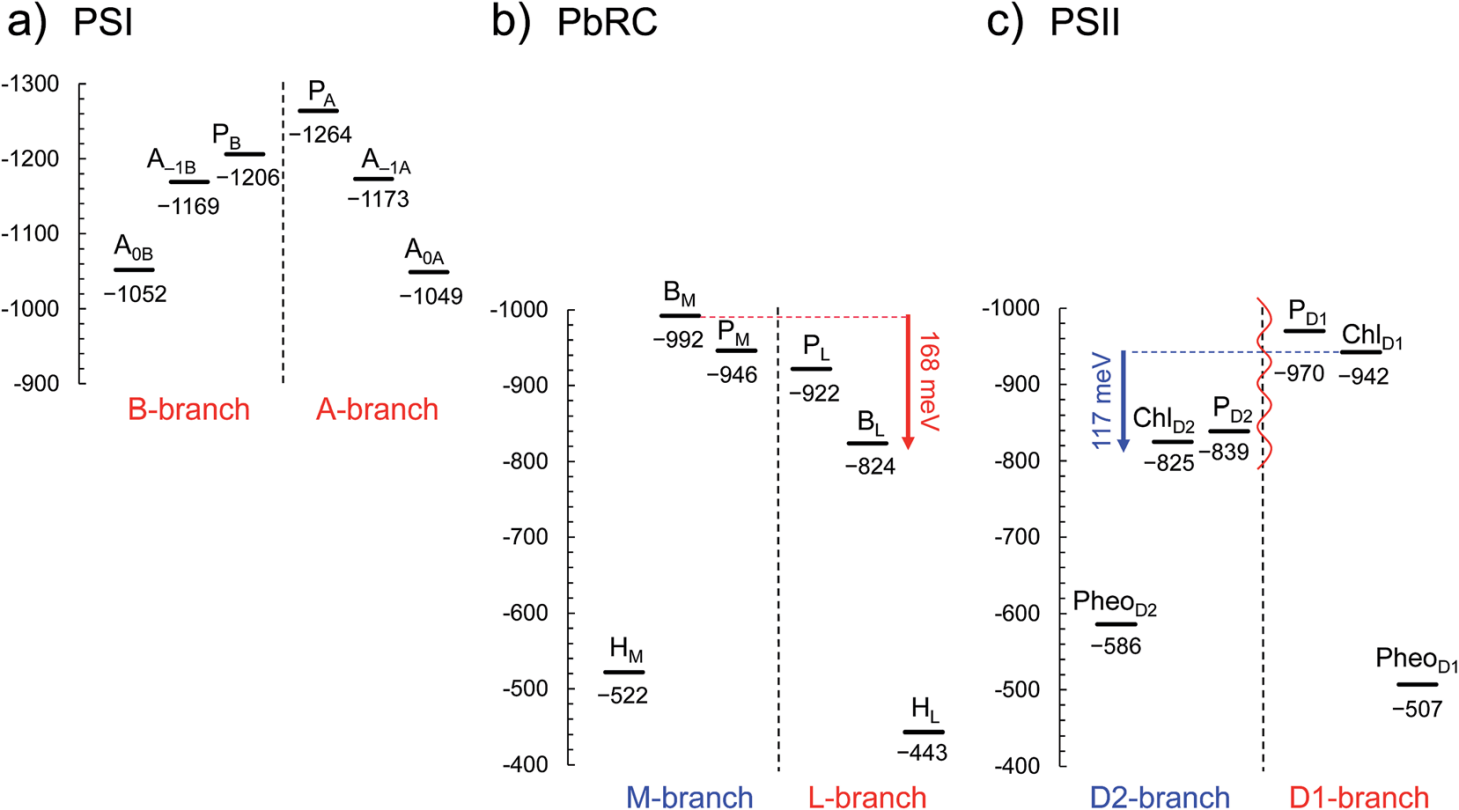
*E*_m_ for one-electron reduction in the electron transfer chains in photosynthetic reaction centers of (a) PSI (PDB code ; 1JB0), (b) PbRC (PDB code ; 3I4D), and (c) PSII (PDB code ; 3ARC) in mV. Red and blue arrows indicate the *E*_m_ difference between accessory (B)Chl*a* cofactors in PbRC and PSII, respectively. The red wavy line indicates the weak electronic coupling (*i.e.*, uncoupling) between P_D1_ and P_D2_.^49^,^50^ Dotted lines indicate pseudo-*C*_2_ axes. Electron-transfer active branches are red labeled, whereas inactive branches are blue labeled. See ref. 13 and 26 for calculated *E*_m_ values for one-electron oxidation in PSI, PbRC, and PSII.

In PSI, *E*_m_(A_–1A_) and *E*_m_(A_–1B_) as well as *E*_m_(A_0A_) and *E*_m_(A_0B_) are at essentially the same level in the cyanobacterial^2^ (Fig. 2a) and plant^6^ (Fig. S1†) PSI crystal structures. *E*_m_(A_0A_) = –1042 mV and *E*_m_(A_0B_) = –1023 mV (Fig. 2a), obtained using the cyanobacterial^2^ PSI crystal structure, are consistent with the experimentally estimated values, *e.g.*, *E*_m_(*A*_0_) = –1050 mV 28 and –1040 mV.^29^

In PbRC, *E*_m_(B_L_) is ∼170 mV less negative than *E*_m_(B_M_) based on the crystal structure analyzed at 2.01 Å resolution (Protein Data Bank (PDB) code ; 3I4D) (Fig. 2b). *E*_m_(B_L_) and *E*_m_(B_M_) were also calculated based on other PbRC crystal structures (*e.g.*, PDB codes, ; 1M3X 3 and ; 1EYS;^30^ Fig. S2†) and show the same tendency.

In sharp contrast to PbRC, *E*_m_(Chl_D1_) is 120 mV more negative than *E*_m_(Chl_D2_) in the 1.9 Å PSII crystal structure^5^ (Fig. 2c). At the Chl_D1_ and Chl_D2_ binding sites, “the PSII protein dielectric volume” (*i.e.*, “uncharged protein volume”, which is ultimately comprised of van der Waals radii of all protein atoms) decreases the solvation of the Chl*a* cofactors, destabilizes Chl*a*˙^–^, and thus lowers the *E*_m_(Chl_D1_) and *E*_m_(Chl_D2_) values. On the other hand, “the atomic charges of proteins” (*i.e.*, “protein charges”) also affect *E*_m_(Chl*a*); *e.g.*, negatively charged groups destabilize Chl*a*˙^–^ and lower the *E*_m_(Chl_D1_) and *E*_m_(Chl_D2_) values. To identify the factors that differentiate between *E*_m_(Chl_D1_) and *E*_m_(Chl_D2_) in PSII, we analyzed contributions of “protein atomic charges” and “loss of solvation” to *E*_m_(Chl_D1_) and *E*_m_(Chl_D2_). Contributions of the protein atomic charges are predominantly responsible for the difference in the *E*_m_ values for accessory chlorophylls between PbRC and PSII, whereas contributions to *E*_m_ from the protein volume, which prevents the solvation of reduced accessory chlorophylls and thus lowers *E*_m_, are much smaller (Table 1).

*E*_m_(Pheo_D1_) is –507 mV (Fig. 2c), which is consistent with the value of –499 mV 31 obtained using the 3.0 Å PSII crystal structure (PDB code ; 2AXT)^32^ and the spectroelectrochemically determined value of –505 mV.^33^

**Table 1:** Contributions of the protein atomic charges and loss of solvation (*i.e.*, due to protein volume, which prevents the solvation of reduced chlorophylls and thus lowers *E*_m_) to *E*_m_ for accessory chlorophylls in mV. The *E*_m_ differences between the two electron-transfer branches are listed in the brackets

Protein	PSI			PbRC			PSII		
Accessory chlorophyll	A_–1B_	A_–1A_		P_M_	P_L_		Chl_D2_	Chl_D1_	
*E*_m_	–1169	–1173	(–4)	–992	–824	(168)	–825	–942	(–117)
In uncharged protein^*a*^	–1085	–1071	(14)	–851	–813	(38)	–1047	–1058	(–11)
In water	–798	–798	(0)	–641	–641	(0)	–798	–798	(0)
*E*_m_ shift (water to protein)	–371	–375	(–4)	–351	–183	(168)	–27	–144	(–117)
Due to protein charge	–84	–102	(–18)	–141	–11	(130)	222	116	(–106)
Due to loss of solvation	–287	–273	(14)	–210	–172	(38)	–249	–260	(–11)

^*a*^Calculated in the absence of all atomic partial charges of the proteins.

### B_L_˙^–^ stabilization in PbRC

**Fig. 3:**
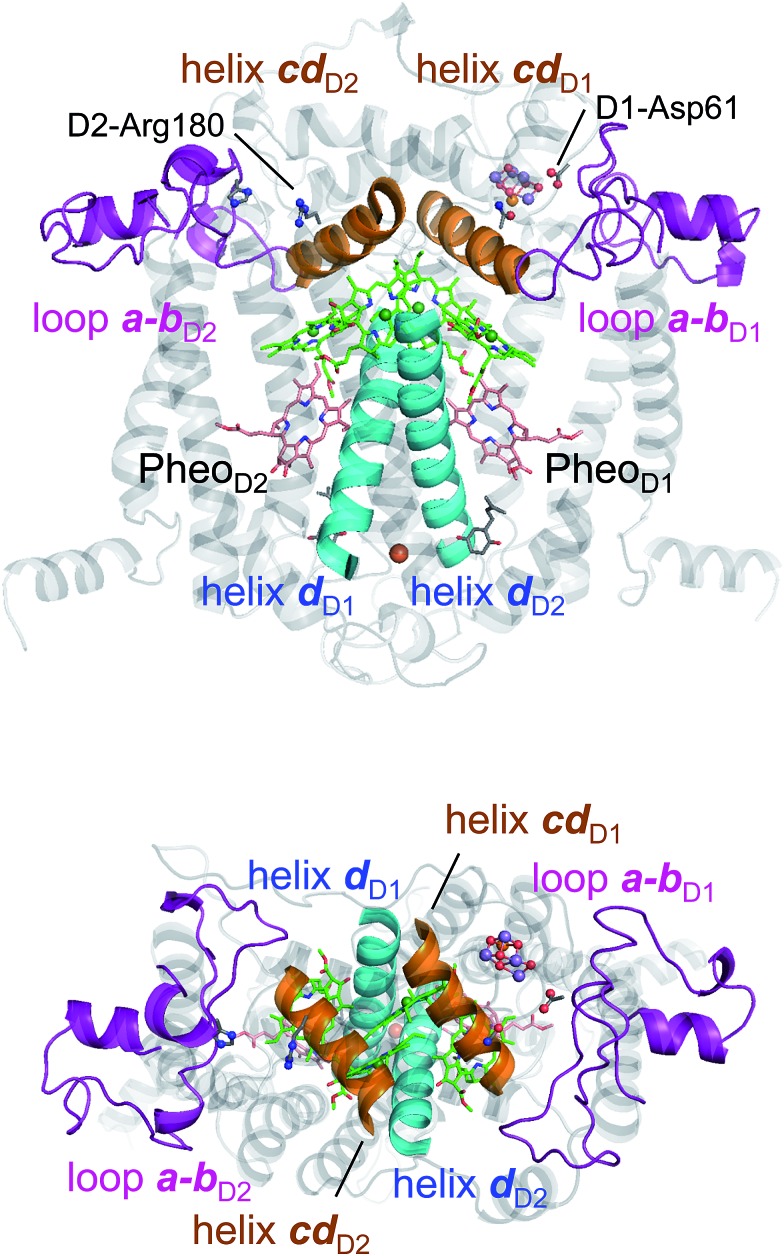
Structural components of type-II reaction centers (*e.g.*, PSII).

In PbRC, electronic excitation of BChl*a* leads to the formation of the (P_L_/P_M_)˙^+^B_L_˙^–^ state.^34^Fig. 2 shows that *E*_m_(B_L_) is 170 mV less negative than *E*_m_(B_M_), facilitating electron transfer along the L-branch. The asymmetry of the electron-transfer energetics is caused by the different contributions of charges on the residues and cofactors, not the different shapes of the proteins (*e.g.*, solvent accessibility near each BChl*a*) (Table 1). In particular, loop *a*–*b* and helix *cd* in the periplasm region and helix *d* in the transmembrane region, which are structurally conserved in PSII (Fig. 3), helped to stabilize B_L_˙^–^ with respect to B_M_˙^–^ (Table 2). Among the L/M-residue pairs, Phe-L181/Tyr-M210 in helix *d* (*E*_m_(B_L_) – *E*_m_(B_M_) = 26 mV), Tyr-L67/Glu-M95 in loop *a–b* (20 mV), and Asp-L155/Asp-M184 in helix *cd* (22 mV) provide the greatest contribution to *E*_m_(B_L_) > *E*_m_(B_M_) (Table 3).

**Table 2:** Contributions of the protein components to *E*_m_ for accessory chlorophylls in PbRC and PSII in mV. —, not applicable

Region	Component	*E*_m_(B_L_) – *E*_m_(B_M_) in PbRC	*E*_m_(Chl_D1_) – *E*_m_(Chl_D2_) in PSII	Difference
Periplasm/lumen	Mn_4_CaO_5_^*a*^	—	56	—
	2Cl^–^	—	–66	—
	Loop *a–b*	47	–86	–133
	Helix *cd*	40	–91	–131
	Others	–7	55	48
Transmembrane	Helix *a*	–7	7	14
	Helix *b*	–3	15	18
	Helix *c*	15	–50	–65
	Helix *d*	50	17	–33
	Helix *e*	–5	26	31
	Cofactors	17	–6	–23
Cytoplasm/stroma	Subunit H	–2	—	—
	Others	9	9	0

^*a*^Including ligand groups.

**Table 3:** Contributions of residues in subunits L and M to *E*_m_(B_L_) and *E*_m_(B_M_) in mV

	*E*_m_(B_L_)	*E*_m_(B_M_)		*E*_m_(B_L_)	*E*_m_(B_M_)	*E*_m_(B_L_) – *E*_m_(B_M_)
Phe-L181	0	22	Tyr-M210	44	–4	26
Val-L157	19	0	Thr-M186	–1	–4	22
Tyr-L67	0	0	Glu-M95	–2	–22	20
Ser-L178	–1	–21	Ala-M207	–7	–2	16
Asp-L155	–21	–5	Asp-M184	–6	–37	15

**Fig. 4:**
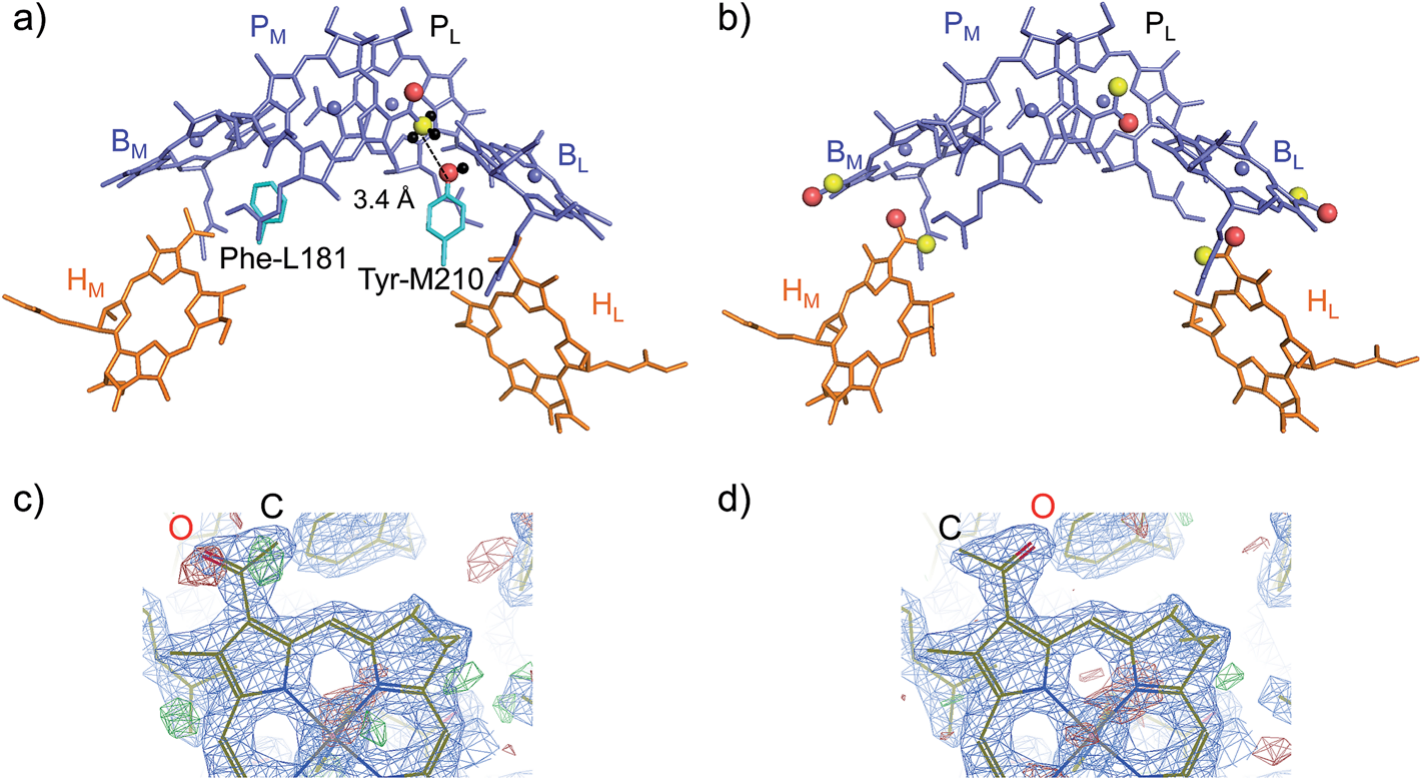
(a) Orientations of the methyl-keto group in P_M_ (yellow ball for methyl C and red ball for keto O) and the hydroxyl group in Tyr-M210 (red ball for hydroxyl O) in the 2.01 Å-PbRC structure (PDB code ; 3I4D). (b) The methyl-keto groups in BChl*a* and BPheo*a* (yellow balls for methyl C and red balls for keto O) in the 1.87 Å-PbRC structure (PDB code ; 2J8C),^4^ whose assignments of the keto O atom and the methyl C atom are opposite to those in the 2.01 Å-PbRC structure (PDB code ; 3I4D). (c) The original assignment of the methyl-keto group of B_L_ in the 1.87 Å-PbRC structure. The density is too low when the keto O atom is assumed (red mesh), whereas too much when the methyl C atom is assumed (green mesh). (d) The swapped assignment of the methyl-keto group of B_L_ in the 1.87 Å-PbRC structure.

(i) *Asp-M184 and Glu-M95 at the binding interface of cytochrome c*_*2*_*, decreasing E*_*m*_*(B*_*M*_*).*Table 3 shows that Glu-M95 and Asp-M184 contribute to decreasing *E*_m_(B_M_) (22 mV and 37 mV, respectively) and thus stabilizing B_L_˙^–^ with respect to B_M_˙^–^. From the observation of the PbRC–cytochrome *c*_2_ co-crystal structure, Axelrod *et al.* concluded that Glu-M95 and Asp-M184 provide the largest electrostatic interaction with cytochrome *c*_2_ (Fig. S3†).^35^ Notably, among the 17 PbRC mutants, mutation of Asp-M184 to Lys exhibits the largest change, with a decrease in the binding constant with cytochrome *c*_2_ by a factor of 800.^36^ Thus, negatively charged Asp-M184 likely contributes not only to binding of the one-electron donor of PbRC (*i.e.*, cytochrome *c*_2_) but also to electron transfer along the L-branch.

(ii) *How Tyr-M210 facilitates L-branch electron transfer*. Among all L/M-residue pairs in PbRC, the difference in the Phe-L181/Tyr-M210 pair in helix *d* contributes to the *E*_m_ difference the most, *i.e.*, increasing *E*_m_(B_L_) with respect to *E*_m_(B_M_), as suggested in theoretical analysis by Parson *et al.*^37^ (Table 3). Indeed, mutations of Tyr-M210 to phenylalanine decreased the initial electron transfer with a time constant from 3.5 ps to 16 ps.^38^ The PbRC crystal structure analyzed at 2.01 Å (PDB code ; 3I4D) shows that the polar –OH group of Tyr-M210 is oriented toward B_L_, thus stabilizing B_L_˙^–^ and increasing *E*_m_(B_L_). The –OH group cannot be oriented toward the methyl-keto (acetyl) group of P_M_ because the methyl site, rather than the keto site, is near the –OH group of Tyr-M210 (Fig. 4a).

In contrast to the 2.01 Å structure, the assignment of the methyl C and keto O atoms of P_M_ is opposite in the PbRC crystal structure analyzed at 1.87 Å (PDB code ; 2J8C);^4^ the methyl-keto orientation allows the –OH group of Tyr-M210 to form an H-bond with the keto O atom of P_M_ (O_PM_–O_TyrM210_ = 3.4 Å; Fig. 4b). Thus, the methyl-keto orientation of P_M_ in the 1.87 Å structure cannot stabilize B_L_˙^–^ (Fig. S4†). However, the electron density map of all BChl*a* and Pheo*a* in the 1.87 Å structure,^4^ except for P_L_, indicates that the density is too low for the keto O atoms (red mesh in Fig. 4c), but too high for the methyl C atoms (green mesh in Fig. 4c) in the original assignment. Remarkably, the swapped assignment of the methyl-keto O and C atoms in P_M_, B_L_, B_M_, H_L_, and H_M_ in the 1.87 Å structure (refined 1.87 Å structure), which is consistent with the original assignment in the 2.01 Å structure, is in better agreement with the density with a decrease in an *R*-factor by 0.01% (Fig. 4d). Just by rotating the methyl-keto groups (forming the refined 1.87 Å structure), the *E*_m_ difference, *i.e.*, *E*_m_(P_L_) – *E*_m_(B_L_), can be altered from –8 mV to –72 mV (Fig. S4†).

Hence, the methyl-keto orientations assigned in the 2.01 Å structure (PDB code 3I4D) appear to be relevant to the PbRC conformation; the –OH group of Tyr-M210 is predominantly oriented toward B_L_, stabilizing B_L_˙^–^ and increasing *E*_m_(B_L_).

(iii) *Low dielectric volume near B*_*M*_*provided by spheroidene and the Q*_*B*_*side chain*. Around the Glu-M95/Asp-M184 moiety, approximately 30 hydrophobic residues from subunit M are in van der Waals contact with the carotenoid spheroidene (Fig. S5†).^39^ The electrostatic influence of the negative charges at the Glu-M95/Asp-M184 moiety is likely to be less screened at B_M_ with respect to B_L_, thus destabilizing B_M_˙^–^. For the same reason, the cluster of hydrophobic residues seems also to enhance the polar –OH group of Tyr-M210 to stabilize B_L_˙^–^. Hence, spheroidene, the cluster of hydrophobic residues, and the Q_B_ isoprene side chain (see below) may be the origin of the low effective dielectric constant reported near B_M_ with respect to B_L_ in the Stark effect spectrum^40^ or the significantly small electric field along the M-branch suggested in electrostatic calculations.^24^ It should be noted that there are no water channels identified near Chl_D1_ and Chl_D2_ in the PSII crystal structures.^41^,^42^

(iv) *The Q*_*B*_*isoprene side chain, decreasing specifically E*_*m*_*(B*_*M*_*)*. The isoprene side chain of Q_B_ is oriented toward B_M_ and is partly in van der Waals contact with spheroidene, whereas that of Q_A_ is oriented away from B_L_ (Fig. S5†).

The isoprene side chain of Q_B_ in the PbRC crystal structure analyzed at 2.01 Å (PDB code ; 3I4D) is comprised of 56 C atoms. When the side chain of Q_B_ is shortened to 16 C atoms, as identified in the 1.87 Å PbRC crystal structure (PDB code ; 2J8C),^4^ and the corresponding inner space is filled by water (represented implicitly with the dielectric constant *ε*_w_ = 80), changes in *E*_m_ are predominantly observed at *E*_m_(B_M_) with an increase of 57 mV (Fig. S6b†); this suggests that the isoprene chain of Q_B_ also contributes to the hydrophobic protein environment specifically for B_M_, enhancing electrostatic interactions and destabilizing B_M_˙^–^.

### Factors that are responsible for *E*_m_(Chl_D1_) < *E*_m_(Chl_D2_) in PSII

Table 2 shows that loop *a*–*b* (86 mV) and helix *cd* (91 mV) in the lumen region (Fig. 3) are responsible for *E*_m_(Chl_D1_) < *E*_m_(Chl_D2_) in PSII. Below we describe the key components that contribute to *E*_m_(Chl_D1_) < *E*_m_(Chl_D2_).

(i) *D1-Asp61/D2-His61 pair in loop a*–*b.* In the stromal/lumen region, loop *a–b* (that connects helices *a* and *b*) and helix *cd* (Fig. 3) seem most likely to characterize PSII with respect to PbRC (Table 2). Loop *a–b* is comprised of 55 residues in D1 (D1: 55–109) and 54 residues in D2 (D2: 55–108), which are almost twice as long as that in PbRC (26 residues in subunit L (L: 57–82) and 34 residues in subunit M (M: 79–112)). The region D2-Val55–Ser66 in PSII is structurally absent in PbRC (Fig. S7†). The insertion in PSII involves key residues for water oxidation, *e.g.*, D1-Ile60 (O_2_-exiting pathway^43^), D1-Asp61 (proton transfer pathway^22^,^44^), D1-Glu65 (proton transfer pathway^22^,^45^ and water channel^42^), and D2-His61 (proton transfer pathway for TyrD^44^,^46^,^47^). In particular, the D1-Asp61/D2-His61 pair decreases *E*_m_(Chl_D1_) by 98 mV (Table 4). The corresponding residues and proton transfer pathways are absent in PbRC.

**Table 4:** Contributions of residues in D1 and D2 to *E*_m_(Chl_D1_) and *E*_m_(Chl_D2_) in mV. —, not applicable

	*E*_m_(Chl_D1_)	*E*_m_(Chl_D2_)		*E*_m_(Chl_D1_)	*E*_m_(Chl_D2_)	*E*_m_(Chl_D1_) – *E*_m_(Chl_D2_)
D1-Asp61	–72	–26	D2-His61	23	75	–98
D1-Asn181^*a*^	5	–1	D2-Arg180	44	123	–73
Cl-1^*a*^	–91	–38	—	—	—	–53
D1-Asp170	–75	–30	D2-Phe169	–2	–6	–42
D1-Tyr161	–8	12	D2-Tyr160	5	24	–39
+D1-His190^*b*^			+D2-His189^*b*^			
D1-Glu65	–11	–2	D2-Ser65	–36	–15	–30
+D1-Asn315^*c*^			+D2-Glu312^*c*^			
D1-Glu189	–47	–26	D2-Phe188	2	5	–24
D1-Ser305	1	3	D2-Glu302	–43	–22	–23
D1-Asp59	–32	–11	D2-Tyr59	0	2	–23
D1-Asn301	2	0	D2-Asp297	–50	–29	–20

^*a*^Cl-1 and D1-Asn181 interact directly (Cl^–^···N_D1-Asn181_ = 3.31 Å^5^).

^*b*^D1-Tyr161 and D1-His190 form an H-bond, sharing a proton. D2-Tyr160 and D2-His189 form an H-bond, sharing a proton.

^*c*^D1-Glu65 and D2-Glu312 form an H-bond, sharing a proton.

(ii) *D2-Arg180 in helix cd, specifically increasing E*_*m*_*(Chl*_*D2*_*)*. In PSII, lumenal helix *cd* (D1: 176–190/D2: 176–188 for PSII, Fig. 3) decreases *E*_m_(Chl_D1_) with respect to *E*_m_(Chl_D2_) by 131 mV, whereas in PbRC, lumenal helix *cd* (and L: 152–162/M: 179–192 for PbRC) increases *E*_m_(B_L_) with respect to *E*_m_(B_M_) by 40 mV (Table 2).

In particular, the D1-Asn181/D2-Arg180 pair in helix *cd* decreases *E*_m_(Chl_D1_) by 73 mV with respect to *E*_m_(Chl_D2_) (Table 4). D1-Asn181 also serves as the Cl-1 binding site^5^ in the proton-conducting E65/E312 water channel.^42^ D2-Arg180 is located at the entrance of the proton transfer pathway for TyrD^44^,^46^ and provides the driving force.^47^ Furthermore, the D1-Asn181/A2-Arg180 pair is responsible for a larger P_D1_˙^+^ population than P_D2_˙^+^ (ref. 13), which facilitates electron transfer from substrate water molecules at the Mn_4_CaO_5_ moiety in D1.

(iii) *Influence of Mn*_*4*_*CaO*_*5*_. *E*_m_ values calculated using the Mn-depleted PSII crystal structure^48^ are similar to those obtained using the 1.9 Å PSII crystal structure (Fig. S8†). Calculated protonation states in the Mn-depleted PSII crystal structure show that the ligand residues (D1-Asp170, D1-Glu189, D1-His332, D1-Glu333, the carboxy-terminal D1-Ala344, and CP43-Glu354) and the H-bond partner (D1-His337) are fully protonated, which could compensate for loss of the cationic Mn_4_CaO_5_ cluster (Table S1†). Hence, the inorganic Mn_4_CaO_5_ component itself is not a main factor that determines *E*_m_ and the energetics of electron transfer.^13^,^26^

## Discussion

### Different mechanism of single-branch electron transfer between PbRC and PSII

*E*_m_(B_L_) is 170 mV less negative than *E*_m_(B_M_) in PbRC. In contrast, the corresponding *E*_m_(Chl_D1_) value is 120 mV more negative than *E*_m_(Chl_D2_) in PSII. These controversial *E*_m_ profiles imply that the mechanisms of single-branch electron transfer are different between PbRC and PSII even though both are type-II reaction centers. The initial electron transfer from the P_L_/P_M_ pair to B_L_ is 100 meV downhill in the L-branch and 50 meV uphill in the M-branch (Fig. 2). This energy difference should facilitate L-branch electron transfer. If P_D1_ and P_D2_ could form the strongly coupled P_D1_/P_D2_ special pair and function as an initial electron donor, electronic excitation of the P_D1_/P_D2_ pair might possibly have led to electron transfer in the D2-branch, since *E*_m_(Pheo_D2_) is sufficiently higher than *E*_m_(Chl_D2_) (Fig. 2). However, the electronic coupling between P_D1_ and P_D2_ (85 to 150 cm^–1^ (ref. 49 and 50)) in PSII is much weaker than that between P_L_ and P_M_ (500 to 1000 cm^–1^ (ref. 51)) in PbRC. In addition, the longest wavelength pigment is thought to be Chl_D1_ in PSII.^50^,^52^ Given that Chl_D1_ is the primary electron donor (*i.e.*, Chl*a*, where excitation occurs due to the lowest site-energy) in PSII (*e.g.*,^53^), the calculated *E*_m_ values indicate that electron transfer can occur in the D1-branch because of the sufficiently high *E*_m_(Pheo_D1_) value (–500 mV 31,33 and Fig. 2).

Hence, it seems likely that PSII activates electron transfer in the D1-branch (i) by uncoupling the P_D1_/P_D2_ pair (*i.e.*, making both electron-transfer branches electronically completely isolated) and (ii) by employing Chl_D1_ as the primary electron donor; in contrast, PbRC activates electron transfer in the L-branch by increasing *E*_m_(B_L_) with respect to *E*_m_(B_M_) in the presence of the strongly coupled P_L_/P_M_ pair.

### Influence of the periplasm/lumen region on *E*_m_ for accessory (B)Chl*a*

In PbRC, among the total *E*_m_ difference of 168 mV between B_L_ and B_M_ (where *E*_m_(B_L_) > *E*_m_(B_M_)), 80 mV originates from the periplasm region, namely loop *a–b* (47 mV) and helix *cd* (40 mV); in PSII, among the total *E*_m_ difference of –117 mV between Chl_D1_ and Chl_D2_ (where *E*_m_(Chl_D1_) < *E*_m_(Chl_D2_)), –132 mV originates from the corresponding lumen region, namely loop *a–b* (–86 mV) and helix *cd* (–91 mV) (Table 2). Thus, loop *a–b* and helix *cd* in the periplasm/lumen region primarily contribute to the different *E*_m_ profiles (Fig. 2) for PbRC and PSII.

In PbRC, acidic residues Asp-M184 in helix *cd* and Glu-M95 in loop *a–b*, which contribute to *E*_m_(B_L_) > *E*_m_(B_M_) (Table 3), serve as an H-bond network for the binding of cytochrome *c*_2_, the source of electrons for [P_L_/P_M_]˙^+^. In PSII, basic residues D2-Arg180 in helix *cd* and D2-His61 in loop *a–b*, which contribute to *E*_m_(Chl_D1_) < *E*_m_(Chl_D2_) (Table 4), serve as a proton-conducting H-bond network proceeding from TyrD^44^,^46^,^47^ and also increase the P_D1_˙^+^ population with respect to P_D2_˙^+^ in [P_D1_/P_D2_]˙^+^.^13^

**Fig. 5:**
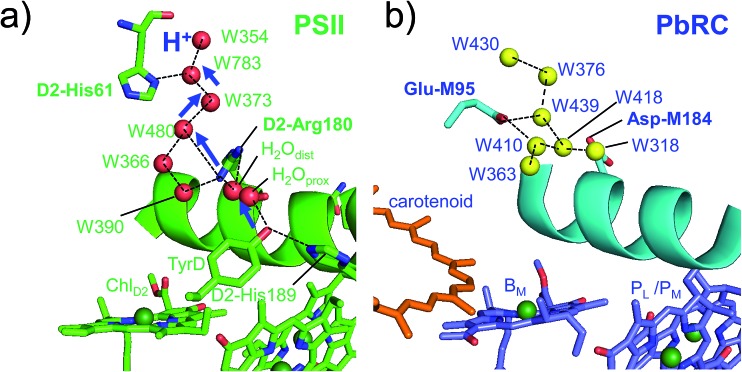
(a) H-bond network of water molecules (red balls) near Chl_D2_ in PSII (green), serving as a proton transfer pathway from TyrD (blue arrows)^44^,^47^ and (b) the corresponding H-bond network of water molecules (yellow balls) near B_M_ in PbRC (cyan). The carotenoid molecule (spheroidene) exists only in PbRC.

Intriguingly, Asp-M184 in helix *cd* and Glu-M95 in loop *a–b* in PbRC correspond to D2-Arg180 in helix *cd* and D2-His61 in loop *a–b* in PSII, respectively (Fig. 5 and S8†). Furthermore, even water molecules in the proton transfer pathway from TyrD in PSII seem to be structurally conserved on the binding surface near Asp-M184 and Glu-M95 in PbRC (Fig. 5b). These structural features imply that the cytochrome *c*_2_ binding network in PbRC and the proton transfer pathway from TyrD in PSII have a common origin, which differentiate the mechanism of single-branch electron transfer between PbRC and PSII.

From the involvement of Asp-M184 in the binding interface with cytochrome *c*_2_ and correspondence of Asp-M184 to D2-Arg180 (Fig. S9†), the electrostatic differences in the periplasm/lumen regions are likely associated with the difference in sources of electrons–cytochrome *c*_2_/H_2_O.

### Type-I reaction centers with respect to type-II reaction centers

In PSII, residues that increase the *E*_m_ difference between Chl_D1_ and Chl_D2_ (Table 2) are mostly identical to those that have been identified to increase the *E*_m_ difference between P_D1_ and P_D2_ significantly^13^ (*e.g.*, D1-Asp61/D2-His61, D1-Asn181/D2-Arg180, D1-Asp170/D2-Phe169, and D1-Glu189/D2-Phe188). These results suggest that the same PSII protein electrostatic environment (discussed above) is responsible for asymmetry in energetics of the electron transfer branches (Fig. 2) as well as the cationic state distribution over the [P_D1_/P_D2_]˙^+^.^13^

In PSI, the protein electrostatic environments of PsaA and PsaB are quite similar and no residues have been identified to induce the *E*_m_ difference between P_A_ and P_B_ significantly.^27^ Indeed, *E*_m_(A_–1A_) and *E*_m_(A_–1B_) are also similar (Fig. 2a and S1†) and there are no residues that induce the *E*_m_ difference between A_–1A_ and A_–1B_. It seems likely that the similar protein electrostatic environment of PsaA and PsaB is a main factor that plays a role in keeping both branches open for electron transfer in PSI.

## Concluding remarks

**Fig. 6:**
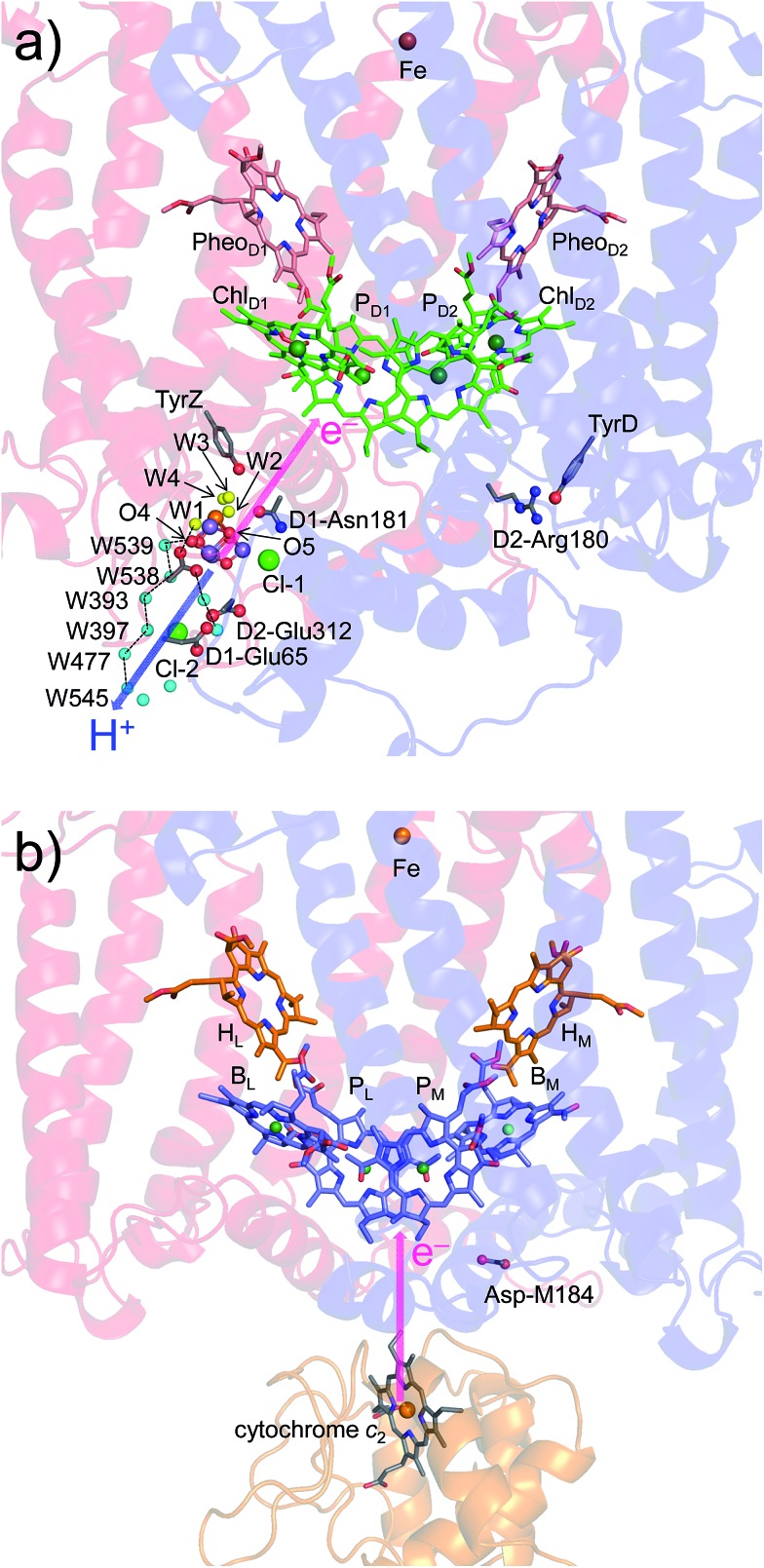
Locations of the electron transfer (pink arrows) and proton transfer (blue arrow) pathways in (a) PSII and (b) PbRC. Ligand water molecules are indicated by yellow balls and other water molecules by cyan balls. PbRCs from *Rhodobacter sphaeroides* and *Blastochloris viridis* have cytochrome *c*_2_ and a bound tetraheme cytochrome as the source of electrons, respectively. In both PbRCs, the sources of electrons are at an equidistance of 20–21 Å from P_L_ and P_M_. In PSII, W539 (21.4 Å), O4 (19.7 Å), and W1 (18.5 Å) are at similar distances (∼20 Å) from the electron acceptor (monomeric P_D1_˙^+^).

In PSII, substrate water molecules need to release protons when acting as an electron donor; thus, both electron and proton transfer pathways are expected to proceed from the substrate water molecules. The proton transfer pathway from O4 and the electron transfer pathway toward P_D1_˙^+^ go along the same axis in the opposite directions (Fig. 6), which allows PSII to use the common protein electrostatic environment for both transfer of electrons (e^–^) and protons (H^+^) without competing. It seems plausible that *E*_m_(Chl_D1_) < *E*_m_(Chl_D2_) in PSII, which is obviously inconsistent with *E*_m_(B_L_) > *E*_m_(B_M_) in PbRC, is due to a compromise between release of protons and release of electrons from the substrate water molecules using the common protein electrostatic environment and could have been overcome (i) by uncoupling the P_D1_/P_D2_ pair and (ii) by employing Chl_D1_ as the primary electron donor.

Hence, it is likely not a coincidence that the D1/D2 residue pairs, which are responsible for *E*_m_(Chl_D1_) < *E*_m_(Chl_D2_) (*e.g.*, D1-Asn181/D2-Arg180 and D1-Asp61/D2-His61), can also serve as (i) electrostatically pushing the cation onto P_D1_ [basic residues in D2],^14^ providing a larger P_D1_˙^+^ population than P_D2_˙^+^ (ref. 13) and thereby facilitating electron transfer from substrate water molecules in D1.^15^ The presence of low p*K*_a_ groups (*i.e.*, acidic residues) near the proton releasing Mn_4_CaO_5_ site [acidic residues in D1] also (ii) facilitates release of protons from the substrate water molecules. These features seem to be the nature of PSII, which uses a protonated electron source—a pair of water molecules.

## Methods

### Coordinates and atomic partial charges

The atomic coordinates were taken from the X-ray structures; cyanobacterial PSI from *Thermosynechococcus elongatus* at 2.5 Å resolution (PDB code, ; 1JB0);^2^ plant PSI from *Pisum sativum* at 2.8 Å resolution (PDB code, ; 4XK8); PbRC from *Rhodobacter sphaeroides* at 2.01 Å resolution (PDB code, ; 3I4D), 1.87 Å resolution (PDB code, ; 2J8C),^4^ and 2.55 Å resolution (PDB code, ; 1M3X);^3^ PbRC from *Thermochromatium tepidum* at 2.2 Å resolution (PDB code, ; 1EYS);^30^ the PSII monomer unit (designated monomer A) of the PSII complexes from *Thermosynechococcus vulcanus* at 1.9 Å resolution (PDB code, ; 3ARC).^5^ Hydrogen atoms were generated and energetically optimized with CHARMM.^54^ Atomic partial charges of the amino acids were adopted from the all-atom CHARMM22) parameter set.^55^ For PSI, the atomic charges of cofactors were taken from previous studies (Chl*a*, phylloquinone, β-carotene,^56^ and the Fe_4_S_4_ cluster^57^). The atomic charges of the other cofactors ((B)Chl*a*, including (B)Chl*a*˙^+^ and (B)Chl*a*˙^–^, (B)Pheo*a*, ubiquinone, plastoquinone, spheroidene, sulfoquinovosyl diacylglycerol, heptyl 1-thiohexopyranoside, and the Fe complex) were determined by fitting the electrostatic potential in the neighborhood of these molecules using the RESP procedure^58^ (Tables S2–S11†). To obtain the atomic charges of the Mn_4_CaO_5_ cluster or the Fe complex, backbone atoms are not included in the RESP procedure (except for D1-Ala344) (Table S11†). The electronic wave functions were calculated after geometry optimization by the DFT method with the B3LYP functional and 6-31G** basis sets, using the JAGUAR program.^59^ For the atomic charges of the non-polar CH_*n*_ groups in cofactors (*e.g.*, the phytol chains of (B)Chl*a* and (B)Pheo*a* and the isoprene side-chains of quinones), the value of +0.09 was assigned for non-polar H atoms. We considered the Mn_4_CaO_5_ cluster to be fully deprotonated in S_1_.

The protein inner spaces were represented implicitly with the dielectric constant *ε*_w_ = 80, whereas the following water molecules were represented explicitly; (i) for PSII, ligand water molecules of the Mn_4_CaO_5_ cluster (W1 to W4), a diamond-shaped cluster of water molecules near TyrZ (W5 to W7)^60^, the water molecule distal to TyrD^44^, ligand water molecules of Chl_D1_ (A1003 and D424), Chl_D2_ (A1009 and A359), and other Chl*a* (B1001, B1007, B1027, C816, and C1004); (ii) for PSI, clusters of water molecules near A_1A_ (A5007, A5015, A5022, A5043, and A5049) and A_1B_ (B5018, B5019, B5030, B5055, B5056, and B5058), ligand water molecules of A_–1A_ (B5005), A_–1B_ (A5005), and other Chl*a* (A5004, A5010, A5012, A5024, A5032, A5051, B5006, B5010, B5022, B5036, B5053, B5054, J127, L4023, and M155).

### *E*_m_ calculation: solving the linear Poisson–Boltzmann equation

To obtain the *E*_m_ values in the proteins, we calculated the electrostatic energy difference between the two redox states in a reference model system by solving the linear Poisson–Boltzmann equation with the MEAD program^61^ and using *E*_m_(BChl*a*) = –641 mV, *E*_m_(BPheo*a*) = –384 mV (based on *E*_m_(BChl*a*) = –830 mV and *E*_m_(BPheo*a*) = –600 mV for one-electron reduction measured in tetrahydrofuran,^62^ considering the solvation energy difference), *E*_m_(Chl*a*) = –798 mV, and *E*_m_(Pheo*a*) = –641 mV (based on *E*_m_(Chl*a*) = –910 mV and *E*_m_(Pheo*a*) = –700 mV for one-electron reduction measured in butyronitrile^63^). The difference in the *E*_m_ value of the protein relative to the reference system was added to the known *E*_m_ value. The ensemble of the protonation patterns was sampled by the Monte Carlo method with Karlsberg.^64^ The linear Poisson–Boltzmann equation was solved using a three-step grid-focusing procedure at resolutions of 2.5 Å, 1.0 Å, and 0.3 Å. Monte Carlo sampling yielded the probabilities [A_ox_] and [A_red_] of the two redox states of molecule A. *E*_m_ was evaluated using the Nernst equation. A bias potential was applied to obtain an equal amount of both redox states ([A_ox_] = [A_red_]), thereby yielding the redox midpoint potential as the resulting bias potential. To facilitate direct comparisons with previous computational results (*e.g.*,^13^,^26^), identical computational conditions and parameters were used; all computations were performed at 300 K, pH 7.0, and an ionic strength of 100 mM; the dielectric constants were set to *ε*_p_ = 4 inside the protein and *ε*_w_ = 80 for water.

## Conflicts of interest

There are no conflicts to declare.

## Supplementary Material

Supplementary information
