# Supplementary material for: Energetic insights into two electron transfer pathways in light-driven energy-converting enzymes
Source: Chem Sci. 2018 Mar 28;9(17):4083–92. doi: 10.1039/c8sc00424b (PMC5944228; doi:10.1039/c8sc00424b)
Supplement: Supplementary file 1 [file SC-009-C8SC00424B-s001.pdf]

Electronic Supplementary Material (ESI) for Chemical Science.
This journal is © The Royal Society of Chemistry 2018

**SI Tables**

**Table S1.** Protonation states calculated using the Mn-depleted PSII structure (PDB codes 5MX2) [2].

| residue | protonation [$H^+$] |
|---|---|
| **ligand** | |
| D1-Asp170 | 1.0 |
| D1-Glu189 | 1.0 |
| D1-His332 | 1.0 |
| D1-Glu333 | 1.0 |
| D1-Asp342 | 0.0 [a] |
| D1-Ala344 [b] | 1.0 |
| CP43-Glu354 | 1.0 |
| **H-bond partner** | |
| D1-His337 | 1.0 |

[a] D1-Asp342 was ionized but it accepted H-bonds from protonated D1-Glu189 and protonated D1-His337.

[b] The carboxy terminus of the D1 protein.

**Table S2.** Atomic partial charges of Chl*a* and Pheo*a*. –, not applicable.

| | Chl$a^{•+}$ | Chl*a* | Chl$a^{•−}$ | Pheo*a* | Pheo$a^{•−}$ |
|---|---|---|---|---|---|
| MG | 0.878 | 0.821 | 0.747 | – | – |
| CHA | 0.086 | 0.071 | 0.081 | 0.109 | 0.081 |
| CHB | -0.386 | -0.365 | -0.350 | -0.337 | -0.360 |
| HHB | 0.161 | 0.144 | 0.129 | 0.144 | 0.135 |
| CHC | -0.255 | -0.221 | -0.247 | -0.161 | -0.205 |
| HHC | 0.146 | 0.138 | 0.131 | 0.127 | 0.118 |
| CHD | -0.359 | -0.280 | -0.326 | -0.220 | -0.296 |
| HHD | 0.189 | 0.173 | 0.169 | 0.165 | 0.165 |
| NA | -0.345 | -0.311 | -0.302 | -0.241 | -0.247 |
| C1A | 0.043 | -0.008 | -0.062 | -0.081 | -0.133 |
| C2A | 0.081 | 0.062 | 0.074 | 0.217 | 0.255 |
| H2A | 0.061 | 0.050 | 0.040 | 0.023 | 0.011 |
| C3A | 0.061 | 0.080 | 0.076 | 0.174 | 0.190 |
| H3A | 0.072 | 0.050 | 0.033 | 0.006 | -0.021 |
| C4A | 0.318 | 0.233 | 0.180 | 0.153 | 0.100 |
| CMA | -0.419 | -0.457 | -0.479 | -0.418 | -0.373 |
| HMA1 | 0.128 | 0.126 | 0.123 | 0.105 | 0.082 |
| HMA2 | 0.128 | 0.126 | 0.123 | 0.105 | 0.082 |
| HMA3 | 0.128 | 0.126 | 0.123 | 0.105 | 0.082 |
| CAA | -0.051 | 0.028 | 0.074 | -0.037 | 0.025 |
| HAA1 | 0.040 | 0.003 | -0.024 | 0.040 | 0.019 |
| HAA2 | 0.040 | 0.003 | -0.024 | 0.040 | 0.019 |
| CBA | -0.296 | -0.302 | -0.301 | -0.548 | -0.578 |
| HBA1 | 0.069 | 0.073 | 0.077 | 0.144 | 0.136 |
| HBA2 | 0.069 | 0.073 | 0.077 | 0.144 | 0.136 |
| CGA | 0.787 | 0.788 | 0.787 | 0.856 | 0.875 |
| O1A | -0.512 | -0.526 | -0.538 | -0.529 | -0.536 |
| O2A | -0.404 | -0.407 | -0.407 | -0.413 | -0.418 |
| NB | -0.436 | -0.371 | -0.285 | -0.115 | -0.044 |
| HNB | – | – | – | 0.189 | 0.170 |
| C1B | 0.223 | 0.131 | 0.014 | 0.087 | 0.007 |
| C2B | 0.132 | 0.151 | 0.156 | 0.123 | 0.115 |
| C3B | -0.071 | -0.088 | -0.156 | -0.063 | -0.130 |
| C4B | 0.248 | 0.135 | 0.086 | 0.039 | -0.001 |
| CMB | -0.376 | -0.350 | -0.278 | -0.311 | -0.248 |
| HMB1 | 0.128 | 0.105 | 0.071 | 0.100 | 0.070 |
| HMB2 | 0.128 | 0.105 | 0.071 | 0.100 | 0.070 |
| HMB3 | 0.128 | 0.105 | 0.071 | 0.100 | 0.070 |
| CAB | -0.077 | -0.045 | 0.006 | -0.051 | 0.000 |
| HAB | 0.122 | 0.105 | 0.087 | 0.112 | 0.096 |
| CBB | -0.331 | -0.391 | -0.475 | -0.387 | -0.474 |
| HBB1 | 0.174 | 0.163 | 0.147 | 0.164 | 0.148 |

| | | | | | |
|---|---|---|---|---|---|
| HBB2 | 0.174 | 0.163 | 0.147 | 0.164 | 0.148 |
| NC | -0.458 | -0.381 | -0.348 | -0.292 | -0.298 |
| C1C | 0.193 | 0.091 | 0.040 | 0.065 | 0.040 |
| C2C | 0.140 | 0.141 | 0.147 | 0.138 | 0.137 |
| C3C | -0.221 | -0.234 | -0.229 | -0.246 | -0.248 |
| C4C | 0.360 | 0.221 | 0.183 | 0.201 | 0.195 |
| CMC | -0.356 | -0.328 | -0.331 | -0.329 | -0.320 |
| HMC1 | 0.125 | 0.100 | 0.087 | 0.100 | 0.085 |
| HMC2 | 0.125 | 0.100 | 0.087 | 0.100 | 0.085 |
| HMC3 | 0.125 | 0.100 | 0.087 | 0.100 | 0.085 |
| CAC | 0.062 | 0.143 | 0.206 | 0.157 | 0.210 |
| HAC1 | 0.039 | 0.000 | -0.036 | -0.006 | -0.036 |
| HAC2 | 0.039 | 0.000 | -0.036 | -0.006 | -0.036 |
| CBC | -0.186 | -0.197 | -0.182 | -0.178 | -0.185 |
| HBC1 | 0.064 | 0.053 | 0.036 | 0.046 | 0.037 |
| HBC2 | 0.064 | 0.053 | 0.036 | 0.046 | 0.037 |
| HBC3 | 0.064 | 0.053 | 0.036 | 0.046 | 0.037 |
| ND | -0.470 | -0.395 | -0.339 | -0.027 | 0.009 |
| HND | – | – | – | 0.091 | 0.072 |
| C1D | 0.288 | 0.143 | 0.094 | 0.005 | -0.013 |
| C2D | 0.114 | 0.113 | 0.089 | 0.171 | 0.118 |
| C3D | -0.196 | -0.218 | -0.269 | -0.272 | -0.301 |
| C4D | 0.188 | 0.124 | 0.088 | 0.037 | 0.028 |
| CMD | -0.345 | -0.300 | -0.233 | -0.320 | -0.254 |
| HMD1 | 0.129 | 0.098 | 0.061 | 0.105 | 0.071 |
| HMD2 | 0.129 | 0.098 | 0.061 | 0.105 | 0.071 |
| HMD3 | 0.129 | 0.098 | 0.061 | 0.105 | 0.071 |
| CAD | 0.629 | 0.627 | 0.618 | 0.661 | 0.641 |
| OBD | -0.424 | -0.473 | -0.535 | -0.481 | -0.542 |
| CBD | -0.624 | -0.630 | -0.653 | -0.618 | -0.642 |
| HBD | 0.217 | 0.207 | 0.197 | 0.213 | 0.207 |
| CGD | 0.840 | 0.859 | 0.870 | 0.763 | 0.786 |
| O1D | -0.529 | -0.536 | -0.547 | -0.508 | -0.536 |
| O2D | -0.379 | -0.398 | -0.407 | -0.338 | -0.342 |
| CED | 0.032 | 0.068 | 0.106 | 0.019 | 0.064 |
| HED1 | 0.076 | 0.055 | 0.032 | 0.067 | 0.044 |
| HED2 | 0.076 | 0.055 | 0.032 | 0.067 | 0.044 |
| HED3 | 0.076 | 0.055 | 0.032 | 0.067 | 0.044 |
| C1 | 0.083 | 0.094 | 0.109 | 0.103 | 0.102 |
| H1 | 0.079 | 0.066 | 0.052 | 0.060 | 0.046 |
| H2 | 0.079 | 0.066 | 0.052 | 0.060 | 0.046 |

**Table S3.** Atomic partial charges of BChl*a* and BPheo*a*. –, not applicable.

| | **BChl*a*$^{\bullet+}$** | **BChl*a*** | **BChl*a*$^{\bullet-}$** | **BPheo*a*** | **BPheo*a*$^{\bullet-}$** |
|---|---|---|---|---|---|
| MG | 0.872 | 0.816 | 0.763 | – | – |
| CHA | 0.100 | 0.089 | 0.097 | 0.150 | 0.117 |
| CHB | -0.410 | -0.378 | -0.408 | -0.400 | -0.448 |
| HHB | 0.157 | 0.144 | 0.135 | 0.151 | 0.143 |
| CHC | -0.444 | -0.389 | -0.429 | -0.387 | -0.426 |
| HHC | 0.201 | 0.180 | 0.172 | 0.180 | 0.170 |
| CHD | -0.445 | -0.404 | -0.396 | -0.364 | -0.412 |
| HHD | 0.185 | 0.169 | 0.151 | 0.168 | 0.158 |
| NA | -0.243 | -0.203 | -0.218 | -0.230 | -0.265 |
| C1A | -0.025 | -0.085 | -0.130 | -0.047 | -0.056 |
| C2A | -0.066 | -0.043 | -0.051 | -0.051 | -0.078 |
| H2A | 0.100 | 0.087 | 0.083 | 0.080 | 0.082 |
| C3A | 0.247 | 0.273 | 0.295 | 0.232 | 0.264 |
| H3A | 0.019 | -0.007 | -0.029 | -0.009 | -0.034 |
| C4A | 0.189 | 0.090 | 0.060 | 0.178 | 0.164 |
| CMA | -0.429 | -0.412 | -0.401 | -0.384 | -0.367 |
| HMA1 | 0.126 | 0.108 | 0.095 | 0.100 | 0.085 |
| HMA2 | 0.126 | 0.108 | 0.095 | 0.100 | 0.085 |
| HMA3 | 0.126 | 0.108 | 0.095 | 0.100 | 0.085 |
| CAA | -0.042 | -0.016 | 0.094 | 0.021 | 0.099 |
| HAA1 | 0.069 | 0.058 | 0.025 | 0.044 | 0.023 |
| HAA2 | 0.069 | 0.058 | 0.025 | 0.044 | 0.023 |
| CBA | -0.410 | -0.437 | -0.488 | -0.442 | -0.497 |
| HBA1 | 0.132 | 0.130 | 0.132 | 0.129 | 0.133 |
| HBA2 | 0.132 | 0.130 | 0.132 | 0.129 | 0.133 |
| CGA | 0.755 | 0.735 | 0.721 | 0.743 | 0.738 |
| O1A | -0.506 | -0.479 | -0.473 | -0.486 | -0.473 |
| O2A | -0.379 | -0.382 | -0.379 | -0.385 | -0.389 |
| NB | -0.524 | -0.453 | -0.419 | -0.232 | -0.200 |
| HNB | – | – | – | 0.209 | 0.205 |
| C1B | 0.299 | 0.193 | 0.152 | 0.161 | 0.129 |
| C2B | 0.164 | 0.174 | 0.140 | 0.161 | 0.116 |
| C3B | -0.374 | -0.432 | -0.510 | -0.428 | -0.499 |
| C4B | 0.438 | 0.350 | 0.355 | 0.298 | 0.292 |
| CMB | -0.352 | -0.319 | -0.253 | -0.299 | -0.222 |
| HMB1 | 0.122 | 0.096 | 0.063 | 0.095 | 0.059 |
| HMB2 | 0.122 | 0.096 | 0.063 | 0.095 | 0.059 |
| HMB3 | 0.122 | 0.096 | 0.063 | 0.095 | 0.059 |
| CAB | 0.686 | 0.715 | 0.735 | 0.726 | 0.741 |
| CBB | -0.509 | -0.518 | -0.524 | -0.532 | -0.535 |
| HBB1 | 0.150 | 0.136 | 0.121 | 0.141 | 0.124 |
| HBB2 | 0.150 | 0.136 | 0.121 | 0.141 | 0.124 |

| | | | | | |
|---|---|---|---|---|---|
| HBB3 | 0.150 | 0.136 | 0.121 | 0.141 | 0.124 |
| OBB | -0.469 | -0.508 | -0.563 | -0.504 | -0.561 |
| NC | -0.307 | -0.235 | -0.244 | -0.213 | -0.243 |
| C1C | 0.190 | 0.058 | 0.035 | 0.102 | 0.071 |
| C2C | 0.198 | 0.282 | 0.293 | 0.265 | 0.314 |
| H2C | 0.026 | -0.010 | -0.017 | -0.008 | -0.029 |
| C3C | -0.033 | -0.016 | -0.055 | -0.073 | -0.086 |
| H3C | 0.064 | 0.037 | 0.034 | 0.046 | 0.030 |
| C4C | 0.232 | 0.116 | 0.081 | 0.148 | 0.141 |
| CMC | -0.303 | -0.295 | -0.291 | -0.297 | -0.278 |
| HMC1 | 0.090 | 0.072 | 0.062 | 0.073 | 0.056 |
| HMC2 | 0.090 | 0.072 | 0.062 | 0.073 | 0.056 |
| HMC3 | 0.090 | 0.072 | 0.062 | 0.073 | 0.056 |
| CAC | -0.028 | -0.015 | 0.006 | 0.020 | 0.032 |
| HAC1 | 0.036 | 0.025 | 0.023 | 0.016 | 0.012 |
| HAC2 | 0.036 | 0.025 | 0.023 | 0.016 | 0.012 |
| CBC | -0.185 | -0.180 | -0.196 | -0.190 | -0.190 |
| HBC1 | 0.063 | 0.047 | 0.040 | 0.049 | 0.036 |
| HBC2 | 0.063 | 0.047 | 0.040 | 0.049 | 0.036 |
| HBC3 | 0.063 | 0.047 | 0.040 | 0.049 | 0.036 |
| ND | -0.513 | -0.462 | -0.403 | -0.092 | -0.060 |
| HND | – | – | – | 0.104 | 0.089 |
| C1D | 0.372 | 0.261 | 0.177 | 0.140 | 0.108 |
| C2D | 0.077 | 0.080 | 0.062 | 0.090 | 0.053 |
| C3D | -0.181 | -0.242 | -0.298 | -0.233 | -0.300 |
| C4D | 0.171 | 0.136 | 0.116 | 0.017 | 0.026 |
| CMD | -0.311 | -0.288 | -0.228 | -0.271 | -0.204 |
| HMD1 | 0.122 | 0.097 | 0.062 | 0.095 | 0.060 |
| HMD2 | 0.122 | 0.097 | 0.062 | 0.095 | 0.060 |
| HMD3 | 0.122 | 0.097 | 0.062 | 0.095 | 0.060 |
| CAD | 0.612 | 0.651 | 0.666 | 0.667 | 0.687 |
| OBD | -0.425 | -0.481 | -0.543 | -0.479 | -0.544 |
| CBD | -0.589 | -0.600 | -0.680 | -0.636 | -0.679 |
| HBD | 0.213 | 0.188 | 0.196 | 0.198 | 0.191 |
| CGD | 0.832 | 0.823 | 0.852 | 0.839 | 0.844 |
| O1D | -0.529 | -0.525 | -0.538 | -0.528 | -0.536 |
| O2D | -0.350 | -0.370 | -0.386 | -0.365 | -0.381 |
| CED | -0.006 | 0.063 | 0.080 | 0.029 | 0.068 |
| HED1 | 0.081 | 0.051 | 0.037 | 0.060 | 0.040 |
| HED2 | 0.081 | 0.051 | 0.037 | 0.060 | 0.040 |
| HED3 | 0.081 | 0.051 | 0.037 | 0.060 | 0.040 |
| C1 | 0.092 | 0.081 | 0.055 | 0.081 | 0.076 |
| H1A | 0.080 | 0.073 | 0.072 | 0.072 | 0.064 |
| H1B | 0.080 | 0.073 | 0.072 | 0.072 | 0.064 |

**Table S4.** Atomic partial charges of ubiquinone (UQ).

| | **UQ** | **$UQ^{\bullet-}$** |
|---|---|---|
| C1 | -0.008 | -0.039 |
| C1M | -0.240 | -0.090 |
| H1MA | 0.090 | 0.018 |
| H1MB | 0.090 | 0.018 |
| H1MC | 0.090 | 0.018 |
| C2 | 0.430 | 0.228 |
| O2 | -0.401 | -0.476 |
| C3 | 0.115 | 0.078 |
| O3 | -0.237 | -0.312 |
| C3M | 0.027 | 0.104 |
| H3MA | 0.059 | 0.010 |
| H3MB | 0.059 | 0.010 |
| H3MC | 0.059 | 0.010 |
| C4 | -0.065 | 0.032 |
| O4 | -0.259 | -0.310 |
| C4M | 0.051 | 0.131 |
| H4MA | 0.048 | 0.002 |
| H4MB | 0.048 | 0.002 |
| H4MC | 0.048 | 0.002 |
| C5 | 0.477 | 0.297 |
| O5 | -0.350 | -0.464 |
| C6 | -0.254 | -0.253 |
| C7 | 0.145 | 0.254 |
| H7A | 0.028 | -0.031 |
| H7B | 0.028 | -0.031 |
| C8 | -0.377 | -0.373 |
| H8 | 0.144 | 0.125 |
| C9 | 0.249 | 0.224 |
| C10 | -0.314 | -0.290 |
| H10A | 0.089 | 0.066 |
| H10B | 0.089 | 0.066 |
| H10C | 0.089 | 0.066 |
| C11 | -0.225 | -0.224 |
| H11A | 0.089 | 0.066 |
| H11B | 0.089 | 0.066 |

**Table S5.** Atomic partial charges of plastoquinone (PLQ).

| | **PLQ** | **PLQ$^{\bullet-}$** |
|---|---|---|
| C2 | -0.352 | -0.402 |
| C3 | -0.082 | -0.171 |
| C4 | 0.480 | 0.324 |
| C5 | -0.067 | -0.084 |
| C6 | -0.103 | -0.122 |
| C1 | 0.558 | 0.442 |
| C7 | 0.264 | 0.448 |
| C8 | -0.467 | -0.487 |
| C9 | 0.294 | 0.243 |
| C10 | -0.373 | -0.336 |
| C11 | -0.268 | -0.254 |
| C52 | -0.130 | -0.099 |
| C53 | -0.152 | -0.053 |
| O1 | -0.437 | -0.557 |
| O2 | -0.427 | -0.575 |
| H2 | 0.160 | 0.139 |
| H7A | 0.007 | -0.061 |
| H7B | 0.007 | -0.061 |
| H8 | 0.163 | 0.165 |
| H10A | 0.106 | 0.081 |
| H10B | 0.106 | 0.081 |
| H10C | 0.106 | 0.081 |
| H11A | 0.104 | 0.081 |
| H11B | 0.104 | 0.081 |
| H52A | 0.065 | 0.023 |
| H52B | 0.065 | 0.023 |
| H52C | 0.065 | 0.023 |
| H53A | 0.068 | 0.009 |
| H53B | 0.068 | 0.009 |
| H53C | 0.068 | 0.009 |

**Table S6.** Atomic partial charges of spheroidene.

| | **spheroidene** |
|---|---|
| CM1 | 0.145 |
| HM1A | 0.015 |
| HM1B | 0.015 |
| HM1C | 0.015 |
| O1 | -0.462 |
| C1 | 0.655 |
| C2 | -0.407 |
| H2A | 0.093 |
| H2B | 0.093 |
| H2C | 0.093 |
| C3 | -0.407 |
| H3A | 0.093 |
| H3B | 0.093 |
| H3C | 0.093 |
| C4 | -0.314 |
| H4A | 0.089 |
| H4B | 0.089 |
| C5 | 0.014 |
| H5 | 0.095 |
| C6 | -0.330 |
| H6 | 0.137 |
| C7 | 0.221 |
| C8 | -0.265 |
| H8A | 0.078 |
| H8B | 0.078 |
| H8C | 0.078 |
| C9 | -0.286 |
| H9 | 0.136 |
| C10 | -0.006 |
| H10 | 0.116 |
| C11 | -0.283 |
| H11 | 0.141 |
| C12 | 0.185 |
| C13 | -0.245 |
| H13A | 0.075 |
| H13B | 0.075 |
| H13C | 0.075 |
| C14 | -0.254 |
| H14 | 0.124 |
| C15 | -0.045 |
| H15 | 0.132 |

| | |
|---|---|
| C16 | -0.250 |
| H16 | 0.149 |
| C17 | 0.209 |
| C18 | -0.336 |
| H18A | 0.095 |
| H18B | 0.095 |
| H18C | 0.095 |
| C19 | -0.259 |
| H19 | 0.130 |
| C20 | -0.102 |
| H20 | 0.121 |
| C21 | -0.078 |
| H21 | 0.125 |
| C22 | -0.264 |
| H22 | 0.127 |
| C23 | 0.214 |
| C24 | -0.243 |
| H24A | 0.073 |
| H24B | 0.073 |
| H24C | 0.073 |
| C25 | -0.317 |
| H25 | 0.141 |
| C26 | 0.014 |
| H26 | 0.122 |
| C27 | -0.285 |
| H27 | 0.144 |
| C28 | 0.061 |
| C29 | -0.255 |
| H29A | 0.083 |
| H29B | 0.083 |
| H29C | 0.083 |
| C30 | -0.114 |
| H30A | 0.050 |
| H30B | 0.050 |
| C31 | 0.215 |
| H31A | 0.000 |
| H31B | 0.000 |
| C32 | -0.399 |
| H32 | 0.135 |
| C33 | 0.099 |
| C34 | -0.286 |
| H34A | 0.088 |
| H34B | 0.088 |
| H34C | 0.088 |
| C35 | -0.132 |

| | |
|---|---|
| H35A | 0.051 |
| H35B | 0.051 |
| C36 | 0.244 |
| H36A | 0.000 |
| H36B | 0.000 |
| C37 | -0.518 |
| H37 | 0.159 |
| C38 | 0.309 |
| C39 | -0.368 |
| H39A | 0.100 |
| H39B | 0.100 |
| H39C | 0.100 |
| C40 | -0.368 |
| H40A | 0.100 |
| H40B | 0.100 |
| H40C | 0.100 |

**Table S7.** Atomic partial charges of sulfoquinovosyl diacylglycerol (SQD).

| | **SQD⁻** |
|---|---|
| O6 | -0.398 |
| C44 | 0.077 |
| C45 | 0.235 |
| C46 | 0.152 |
| O47 | -0.359 |
| C7 | 0.735 |
| O49 | -0.540 |
| C8 | -0.302 |
| O48 | -0.388 |
| C23 | 0.788 |
| O10 | -0.546 |
| C24 | -0.358 |
| C1 | 0.428 |
| C2 | 0.114 |
| O2 | -0.601 |
| C3 | 0.202 |
| O3 | -0.628 |
| C4 | 0.041 |
| O4 | -0.606 |
| C5 | 0.312 |
| C6 | -0.221 |
| O5 | -0.433 |
| S | 1.004 |
| O7 | -0.610 |
| O8 | -0.610 |
| O9 | -0.610 |
| H44A | 0.062 |
| H44B | 0.062 |
| H45 | 0.020 |
| H46A | 0.000 |
| H46B | 0.000 |
| H8A | 0.117 |
| H8B | 0.117 |
| H24A | 0.136 |
| H24B | 0.136 |
| H1 | 0.003 |
| H2 | 0.062 |
| HO | 0.398 |
| H3 | 0.022 |
| HO3 | 0.412 |
| H4 | 0.036 |

| | |
|---|---|
| HO4 | 0.399 |
| H5 | 0.018 |
| H6A | 0.061 |
| H6B | 0.061 |

**Table S8.** Atomic partial charges of heptyl 1-thiohexopyranoside (HTG).

| | **HTG** |
|---|---|
| C1 | 0.039 |
| S1 | -0.219 |
| C2 | 0.274 |
| O2 | -0.609 |
| C3 | 0.072 |
| O3 | -0.618 |
| C4 | 0.145 |
| O4 | -0.601 |
| C5 | 0.361 |
| O5 | -0.415 |
| C6 | 0.064 |
| O6 | -0.600 |
| C1' | 0.008 |
| C2' | -0.015 |
| C3' | 0.098 |
| C4' | -0.175 |
| C5' | -0.047 |
| C6' | 0.139 |
| C7' | -0.275 |
| H1 | 0.112 |
| H2 | 0.062 |
| HO | 0.416 |
| H3 | 0.054 |
| HO3 | 0.426 |
| H4 | 0.042 |
| HO4 | 0.393 |
| H5 | -0.038 |
| H6A | 0.036 |
| H6B | 0.036 |
| HO6 | 0.410 |
| H1'1 | 0.062 |
| H1'2 | 0.062 |
| H2'1 | 0.002 |
| H2'2 | 0.002 |
| H3'1 | -0.002 |
| H3'2 | -0.002 |
| H4'1 | 0.044 |
| H4'2 | 0.044 |
| H5'1 | 0.021 |
| H5'2 | 0.021 |
| H6'1 | -0.009 |

| | |
|---|---|
| H6'2 | -0.009 |
| H7'1 | 0.063 |
| H7'2 | 0.063 |
| H7'3 | 0.063 |

**Table S9.** Atomic partial charges of the Fe complex in PbRC.

| **Fe complex (PbRC)** | |
|---|---|
| 1FE | 0.746 |
| **Glu-M234** | |
| 2CB | -0.081 |
| 2HB1 | 0.036 |
| 2HB2 | 0.036 |
| 2CG | 0.034 |
| 2HG1 | 0.005 |
| 2HG2 | 0.005 |
| 2CD | 0.633 |
| 2OE1 | -0.635 |
| 2OE2 | -0.635 |
| **His-L190** | |
| 3CB | -0.258 |
| 3HB1 | 0.126 |
| 3HB2 | 0.126 |
| 3CG | 0.268 |
| 3ND1 | -0.300 |
| 3HD1 | 0.340 |
| 3CD2 | -0.254 |
| 3HD2 | 0.168 |
| 3CE1 | 0.038 |
| 3HE1 | 0.156 |
| 3NE2 | -0.197 |
| **His-M219** | |
| 4CB | -0.258 |
| 4HB1 | 0.126 |
| 4HB2 | 0.126 |
| 4CG | 0.268 |
| 4ND1 | -0.300 |
| 4HD1 | 0.340 |
| 4CD2 | -0.254 |
| 4HD2 | 0.168 |
| 4CE1 | 0.038 |
| 4HE1 | 0.156 |
| 4NE2 | -0.197 |
| **His-M266** | |
| 5CB | -0.258 |
| 5HB1 | 0.127 |
| 5HB2 | 0.127 |
| 5CG | 0.268 |
| 5ND1 | -0.300 |

| | |
|---|---|
| 5HD1 | 0.340 |
| 5CD2 | -0.254 |
| 5HD2 | 0.168 |
| 5CE1 | 0.038 |
| 5HE1 | 0.156 |
| 5NE2 | -0.197 |
| **His-L190** | |
| 6CB | -0.258 |
| 6HB1 | 0.127 |
| 6HB2 | 0.127 |
| 6CG | 0.268 |
| 6ND1 | -0.300 |
| 6HD1 | 0.340 |
| 6CD2 | -0.254 |
| 6HD2 | 0.168 |
| 6CE1 | 0.038 |
| 6HE1 | 0.156 |
| 6NE2 | -0.197 |

**Table S10.** Atomic partial charges of the Fe complex in PSII.

| **Fe complex (PSII)** | |
|---|---|
| 1FE | 0.695 |
| **$HCO_3^-$** | |
| 2C | 0.847 |
| 2O1 | -0.534 |
| 2O2 | -0.635 |
| 2O3 | -0.635 |
| 2HO | 0.385 |
| **D2-His214** | |
| 3CB | -0.259 |
| 3CG | 0.266 |
| 3ND1 | -0.288 |
| 3CD2 | -0.262 |
| 3CE1 | 0.037 |
| 3NE2 | -0.182 |
| 3HB1 | 0.131 |
| 3HB2 | 0.131 |
| 3HD1 | 0.342 |
| 3HD2 | 0.174 |
| 3HE1 | 0.153 |
| **D1-His215** | |
| 4CB | -0.271 |
| 4CG | 0.328 |
| 4ND1 | -0.330 |
| 4CD2 | -0.356 |
| 4CE1 | 0.009 |
| 4NE2 | -0.090 |
| 4HB1 | 0.130 |
| 4HB2 | 0.130 |
| 4HD1 | 0.347 |
| 4HD2 | 0.188 |
| 4HE1 | 0.181 |
| **D1-His272** | |
| 5CB | -0.258 |
| 5CG | 0.209 |
| 5ND1 | -0.232 |
| 5CD2 | -0.176 |
| 5CE1 | -0.029 |
| 5NE2 | -0.239 |
| 5HB1 | 0.128 |
| 5HB2 | 0.128 |
| 5HD1 | 0.324 |

| | |
|---|---|
| 5HD2 | 0.151 |
| 5HE1 | 0.178 |
| **D2-His268** | |
| 6CB | -0.270 |
| 6CG | 0.273 |
| 6ND1 | -0.336 |
| 6CD2 | -0.229 |
| 6CE1 | 0.114 |
| 6NE2 | -0.252 |
| 6HB1 | 0.130 |
| 6HB2 | 0.130 |
| 6HD1 | 0.346 |
| 6HD2 | 0.159 |
| 6HE1 | 0.119 |

**Table S11.** Atomic partial charges of the $Mn_4CaO_5$ cluster in $S_1$.

| **$Mn_4CaO_5$ cluster** | |
|---|---|
| 1Mn1 | 1.398 |
| 1Mn2 | 1.768 |
| 1Mn3 | 1.904 |
| 1Mn4 | 1.549 |
| 1Ca1 | 1.743 |
| 1O1 | -1.010 |
| 1O2 | -1.148 |
| 1O3 | -0.979 |
| 1O4 | -1.014 |
| 1O5 | -1.071 |
| **water ligand** | |
| 1OH1 | -0.935 |
| 1H11 | 0.482 |
| 1H12 | 0.450 |
| 1OH2 | -0.958 |
| 1H21 | 0.444 |
| 1H22 | 0.511 |
| 1OH3 | -0.862 |
| 1H31 | 0.490 |
| 1H32 | 0.496 |
| 1OH4 | -0.921 |
| 1H41 | 0.458 |
| 1H42 | 0.536 |
| **D1-Asp170** | |
| 2CB | -0.307 |
| 2CG | 0.932 |
| 2OD1 | -0.839 |
| 2OD2 | -0.795 |
| 2HB1 | 0.091 |
| 2HB2 | 0.091 |
| **D1-Glu189** | |
| 3CB | -0.292 |
| 3CG | -0.041 |
| 3CD | 0.757 |
| 3OE1 | -0.696 |
| 3OE2 | -0.715 |
| 3HB1 | 0.085 |
| 3HB2 | 0.085 |
| 3HG1 | 0.081 |
| 3HG2 | 0.081 |
| **D1-His332** | |

| | |
|---|---|
| 4ND1 | -0.294 |
| 4CG | 0.400 |
| 4CB | -0.515 |
| 4NE2 | -0.165 |
| 4CD2 | -0.294 |
| 4CE1 | 0.029 |
| 4HD1 | 0.435 |
| 4HB1 | 0.213 |
| 4HB2 | 0.213 |
| 4HD2 | 0.174 |
| 4HE1 | 0.201 |
| **D1-Glu333** | |
| 5CB | -0.084 |
| 5CG | -0.076 |
| 5CD | 0.784 |
| 5OE1 | -0.658 |
| 5OE2 | -0.652 |
| 5HB1 | 0.064 |
| 5HB2 | 0.064 |
| 5HG1 | 0.045 |
| 5HG2 | 0.045 |
| **D1-Asp342** | |
| 6CB | -0.416 |
| 6CG | 0.927 |
| 6OD1 | -0.737 |
| 6OD2 | -0.780 |
| 6HB1 | 0.192 |
| 6HB2 | 0.192 |
| **D1-Ala344** | |
| 7N | -0.310 |
| 7HN | 0.310 |
| 7CA | -0.016 |
| 7C | 0.658 |
| 7OT1 | -0.634 |
| 7CB | -0.313 |
| 7OT2 | -0.680 |
| 7HA | 0.054 |
| 7HB1 | 0.099 |
| 7HB2 | 0.099 |
| 7HB3 | 0.099 |
| **CP43-Glu354** | |
| 8CB | -0.132 |
| 8CG | -0.185 |
| 8CD | 0.821 |
| 8OE1 | -0.645 |

| | |
|---|---|
| 8OE2 | -0.748 |
| 8HB1 | 0.056 |
| 8HB2 | 0.056 |
| 8HG1 | 0.113 |
| 8HG2 | 0.113 |
| **CP43-Arg357** | |
| 9CB | -0.238 |
| 9CG | -0.033 |
| 9CD | 0.306 |
| 9NE | -0.709 |
| 9CZ | 0.911 |
| 9NH1 | -0.838 |
| 9NH2 | -1.007 |
| 9HB1 | 0.095 |
| 9HB2 | 0.095 |
| 9HG1 | 0.041 |
| 9HG2 | 0.041 |
| 9HD1 | -0.003 |
| 9HD2 | -0.003 |
| 9HE | 0.419 |
| 9HH11 | 0.443 |
| 9HH12 | 0.443 |
| 9HH21 | 0.533 |
| 9HH22 | 0.533 |

**SI Figure legends**

**Figure S1.** $E_m$ for one-electron reduction calculated using a) the cyanobacterial PSI crystal structure (PDB code 1JB0) [3] and b) the plant PSI crystal structure (PDB code 4XK8) [4] in mV.

**Figure S2.** $E_m$ for one-electron reduction calculated using the crystal structures of PbRC from a) *Rhodobacter sphaeroides* (PDB codes 1M3X) [5] and b) *Thermochromatium tepidum* (PDB codes 1EYS) [6] in mV.

**Figure S3.** (a) The binding interface of cytochrome $c_2$ with PbRC [7] and (b) the corresponding region in D1 (yellow) and D2 (blue) of PSII.

**Figure S4.** $E_m$ for one-electron reduction calculated using a) the 1.87 Å-structure (PDB codes 2J8C) [8] and b) the refined 1.87 Å-structure, where the methyl-keto O and C atoms in $P_M$, $B_L$, $B_M$, $H_L$, and $H_M$ in the original 1.87 Å-structure were swapped.

**Figure S5.** Localization of hydrophobic residues (white sphere) and cofactors, spheroidene (red sphere) and $Q_B$ (cyan sphere), at the $B_M$ binding moiety. Hydrophobic residues shown are Trp-M66, Phe-M67, Phe-M68, Phe-M74, Trp-M75, Phe-M85, Phe-M105, Trp-M115, Phe-M120, Phe-M123, Trp-M157, Phe-M162, Trp-M171, Pro-M176, Tyr-M177, and His-M182.

**Figure S6.** a) Deletion of the $Q_B$ isoprene side-chain from $C_{56}$ to $C_{16}$ in the 2.01 Å-PbRC crystal structure (PDB code 3I4D) and b) changes in $E_m$ for one-electron reduction (red arrow) in mV. Note that the $Q_B$ isoprene side-chain is comprised of $C_{16}$ in the 1.87 Å-PbRC crystal structure (PDB code 2J8C).

**Figure S7.** Loop ***a-b*** and helix ***cd*** of D2 in PSII (green) and subunit M in PbRC (cyan). The region between D2-Val55 and Ser66 in PSII (red dotted circle), where D2-His61 [9] is also located, is structurally absent in PbRC.

**Figure S8.** $E_m$ for one-electron reduction calculated using the Mn-depleted PSII crystal structure (PDB codes 5MX2) [2] in mV.

**Figure S9.** The amino acid sequences of the L and M subunits in PbRC and the D1 and D2 subunits in PSII. Asp-M184, D1-Asn181/D2-Arg180, and Phe-L181/Tyr-M210 are in bold.

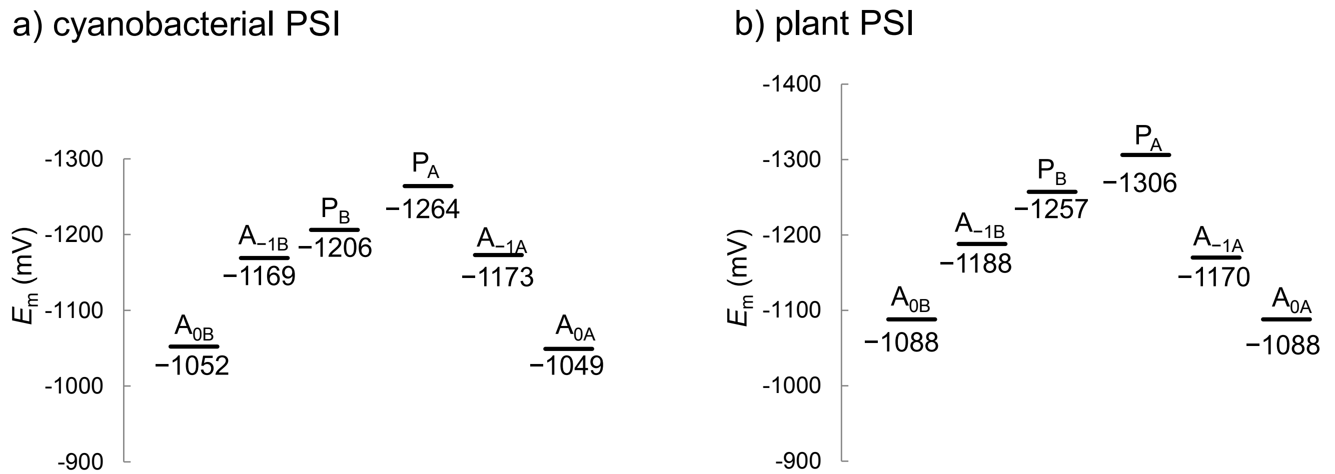


**Figure S1.** $E_m$ for one-electron reduction calculated using a) the cyanobacterial PSI crystal structure (PDB code 1JB0) [3] and b) the plant PSI crystal structure (PDB code 4XK8) [4].

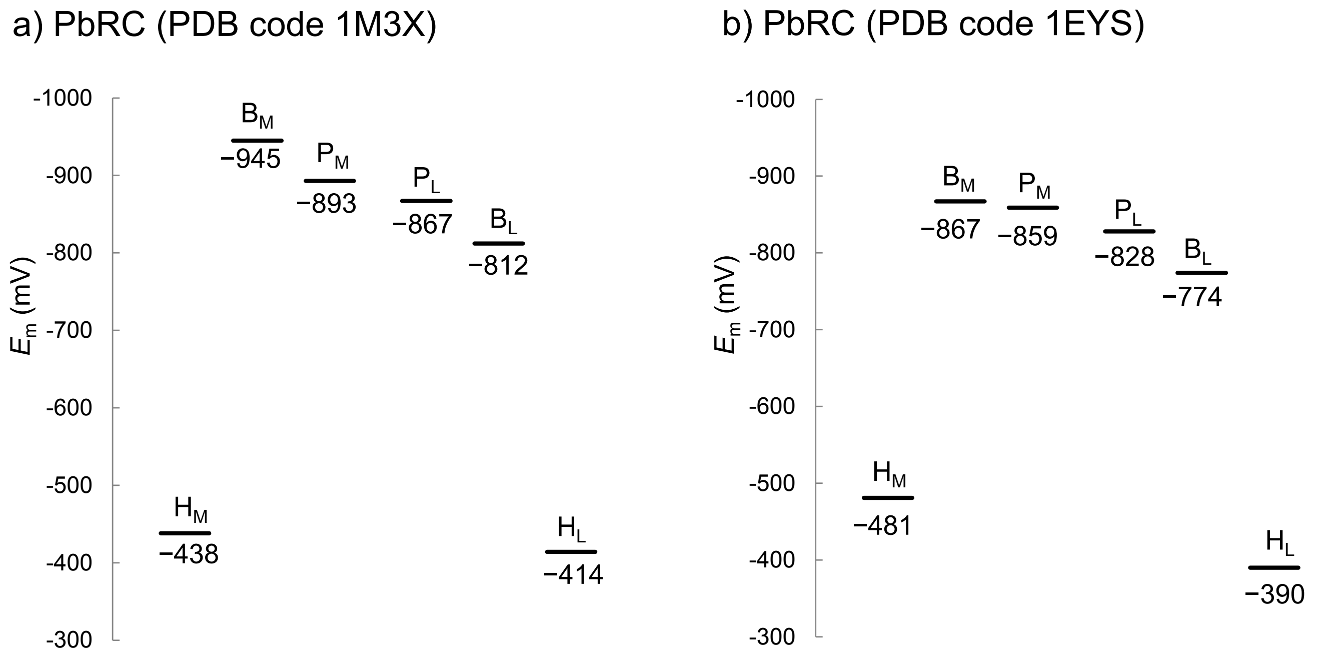


**Figure S2.** $E_m$ for one-electron reduction calculated using the crystal structures of PbRC from a) *Rhodobacter sphaeroides* (PDB codes 1M3X) [5] and b) *Thermochromatium tepidum* (PDB codes 1EYS) [6].

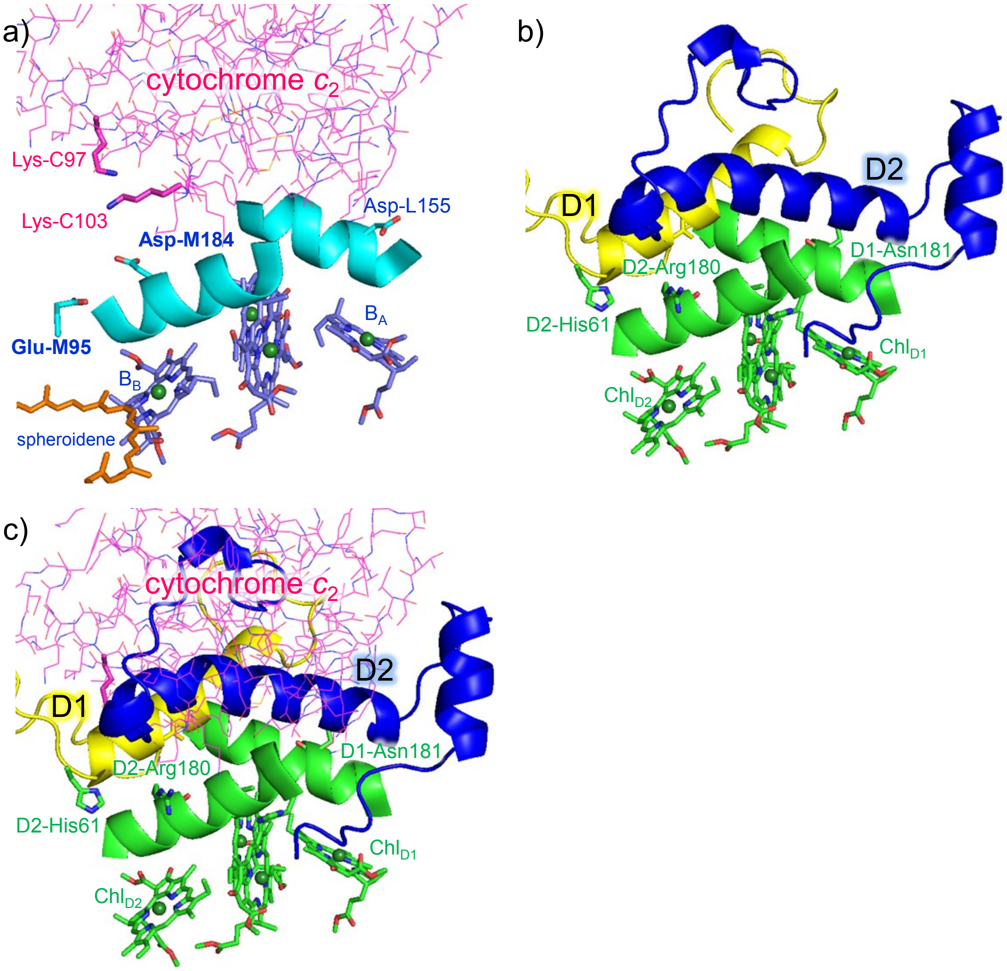


**Figure S3.** (a) The binding interface of cytochrome $c_2$ with PbRC (cyan) [7], (b) the corresponding region in D1 (yellow) and D2 (blue) (where cytochrome $c_2$ would exist) in PSII (green), and (c) comparison between PbRC (cytochrome $c_2$) and PSII (D1 and D2 proteins).

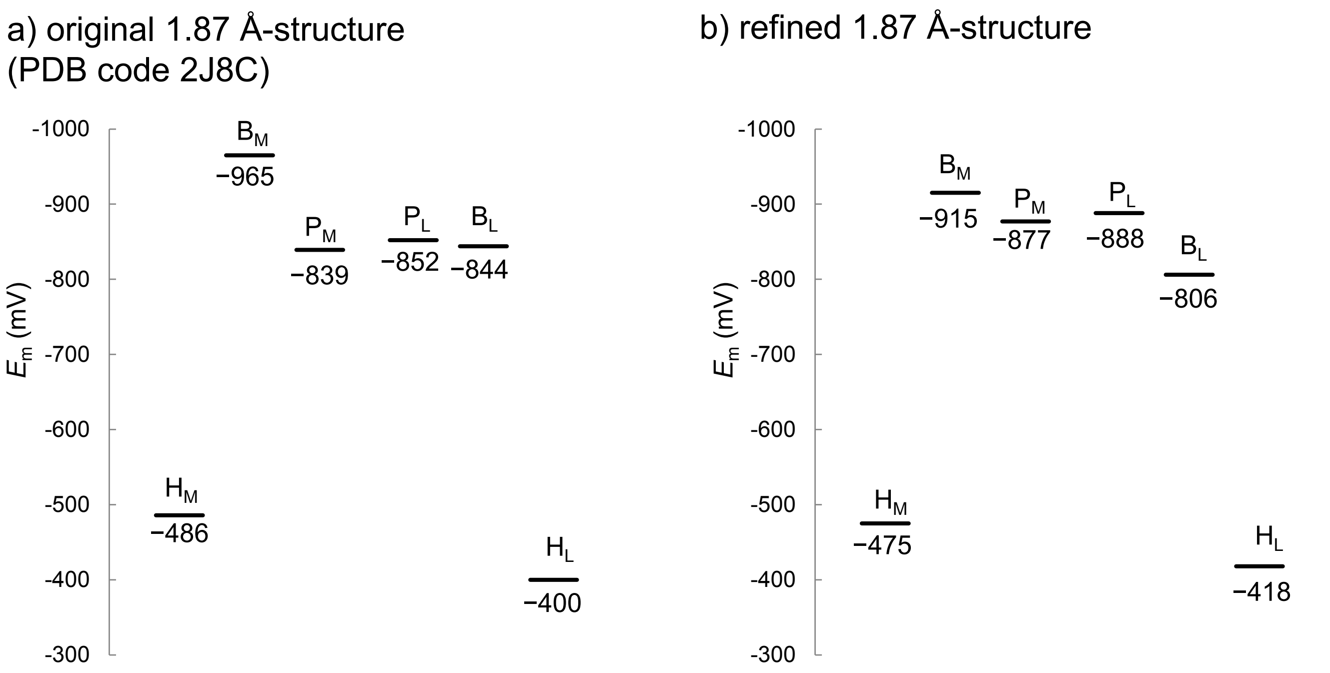


**Figure S4.** $E_m$ for one-electron reduction calculated using a) the 1.87 Å-structure (PDB codes 2J8C) [8] and b) the refined 1.87 Å-structure, where the methyl-keto O and C atoms in $P_M$, $B_L$, $B_M$, $H_L$, and $H_M$ in the original 1.87 Å-structure were swapped.

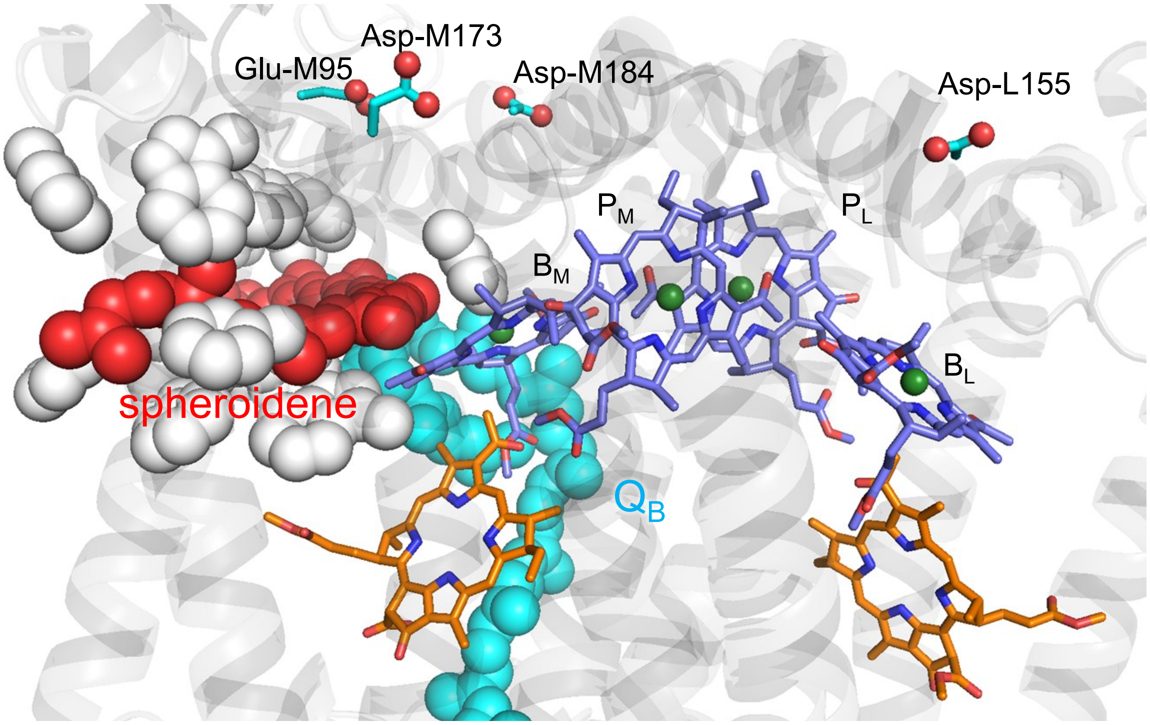


**Figure S5.** Localization of hydrophobic residues (white sphere) and cofactors, spheroidene (red sphere) and $Q_B$ (cyan sphere), at the $B_M$ binding moiety. Hydrophobic residues shown are Trp-M66, Phe-M67, Phe-M68, Phe-M74, Trp-M75, Phe-M85, Phe-M105, Trp-M115, Phe-M120, Phe-M123, Trp-M157, Phe-M162, Trp-M171, Pro-M176, Tyr-M177, and His-M182.

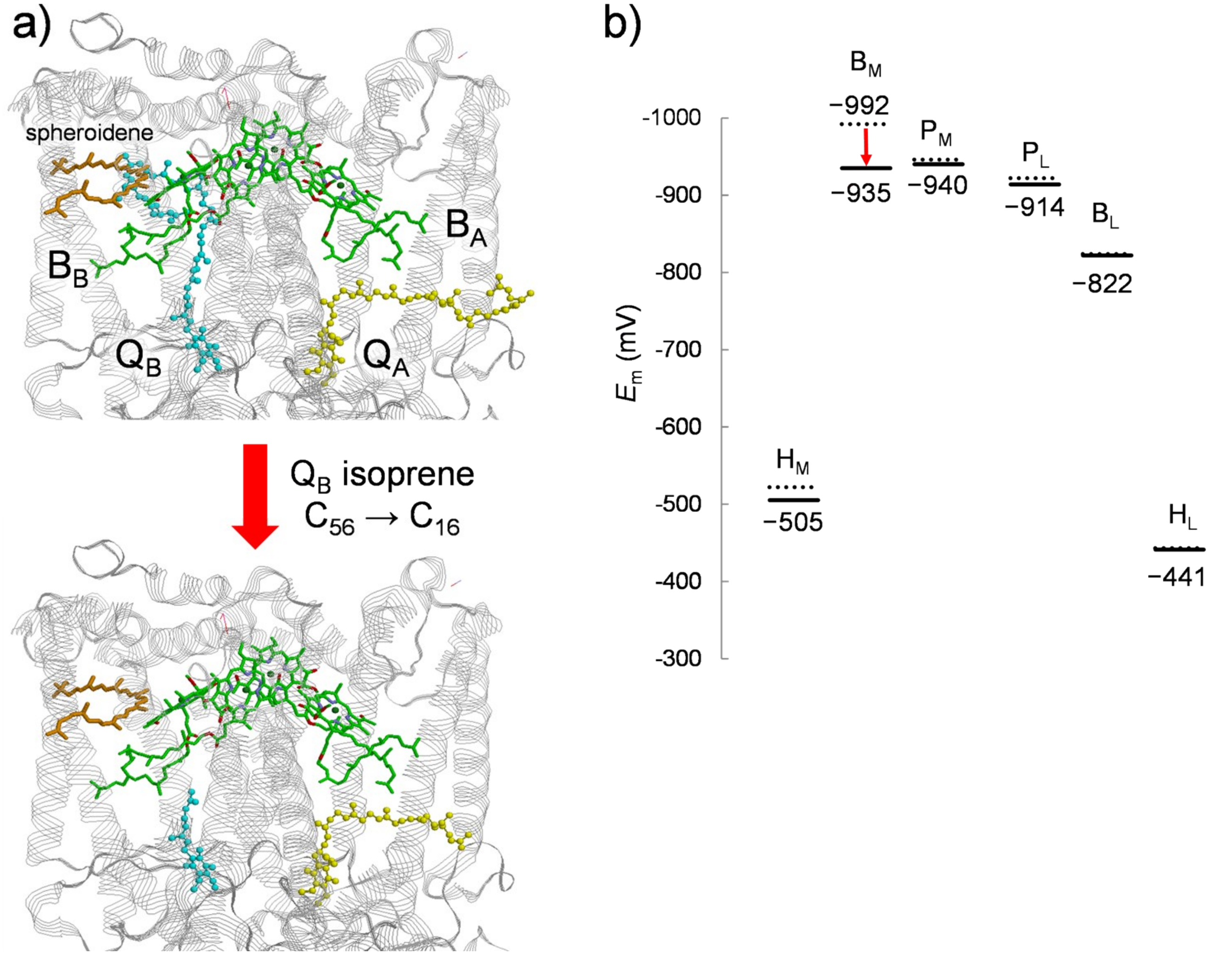


**Figure S6.** a) Deletion of the $Q_B$ isoprene side-chain from $C_{56}$ to $C_{16}$ in the 2.01 Å-PbRC crystal structure (PDB code 3I4D) and b) changes in $E_m$ for one-electron reduction (red arrow) in mV. Note that the $Q_B$ isoprene side-chain is comprised of $C_{16}$ in the 1.87 Å-PbRC crystal structure (PDB code 2J8C).

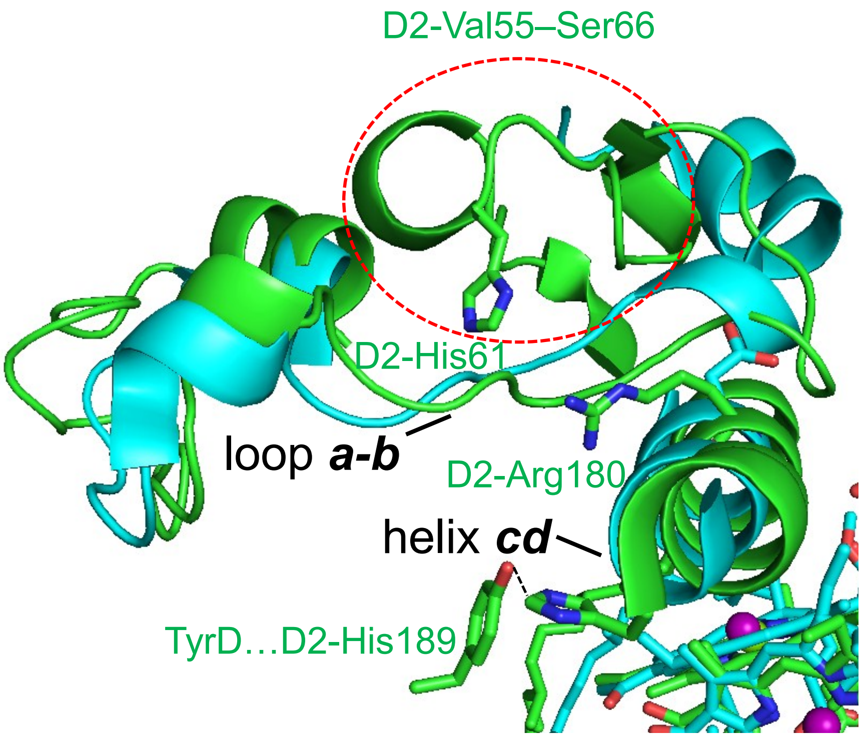


**Figure S7.** Loop ***a-b*** and helix ***cd*** of D2 in PSII (green) and subunit M in PbRC (cyan). The region between D2-Val55 and Ser66 in PSII (red dotted circle), where D2-His61 [9] is also located, is structurally absent in PbRC.

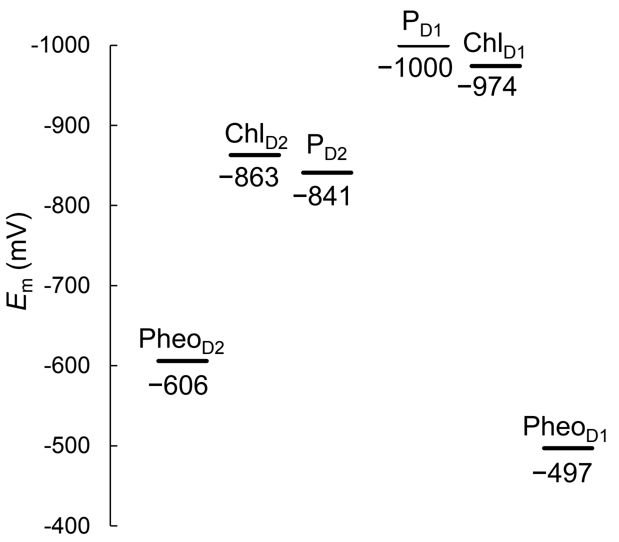


**Figure S8.** $E_m$ for one-electron reduction calculated using the Mn-depleted PSII crystal structure (PDB codes 5MX2) [2] in mV.

```
                                                 Asp-M184,D1-Asn181/D2-Arg180
                                                  ↓                         ↓Phe-L181/Tyr-M210
L-R.sphaeroides       ayltlvlfrpvmmgawgyafpygiwthldwvsntgytygnfhynpahmiaisffftnala
L-T.tepidum           aylvlvfvrpllmgawghgfpygilshldwvsnvgyqflhfhynpahmlaisffftncla
L-B.viridis           mfcvlqvfrplllgswghafpygilshldwvnnfgyqylnwhynpghmssvsflfvnama
----------------------------------------------------------------------------------
M-R.sphaeroides       lwmvlgfirpilmgswseavpygifshldwtnnfslvhgnlfynpfhglsiaflygsall
M-T.tepidum           fylvlgfirpvmmgswakavpfgifphldwtaafsirygnlyynpfhmlsiaflygsall
M-B.viridis           fvlcigcihptlvgswsegvpfgiwphidwltafsirygnfyycpwhgfsigfaygcgll
----------------------------------------------------------------------------------
D1-T.elongatus        safavfliypigqgsfsdgmplgisgtfnfmivfq-aehnilmhpfhqlgvagvfggalf
D1-C.caldarium        aatavfiiypigqgsfsdgmplgisgtfnfmlvfq-aehnilmhpfhmmgvagvfggslf
D1-C.reinhardtii      aasavflvypigqgsfsdgmplgisgtfnfmivfq-aehnilmhpfhmlgvagvfggslf
D1-spinach            aatavfliypigqgsfsdgmplgisgtfnfmivfq-aehnilmhpfhmlgvagvfggslf
----------------------------------------------------------------------------------
D2-T.elongatus        vfvsvfliyplgqsswffapsfgvaaifrfllffq-gfhnwtlnpfhmmgvagvlggall
D2-C.caldarium        vfvsvfliyplgqaswffapsfgvaaifrfilflq-gfhnwtlnpfhmmgvagilggall
D2-C.reinhardtii      vfvsvfliyplgqsgwffapsfgvaaifrfilffq-gfhnwtlnpfhmmgvagvlgaall
D2-spinach            vfvsvfliyplgqsgwffapsfgvaaifrfilffq-gfhnwtlnpfhmmgvagvlgaall
                          :  . *   ..:  .   *:   : :         :    * *  .:.      :
```

**Figure S9.** The amino acid sequences of the L and M subunits in PbRC and the D1 and D2 subunits in PSII. Asp-M184, D1-Asn181/D2-Arg180, and Phe-L181/Tyr-M210 are in bold. Asterisks (*) indicate a fully conserved site. Colons (:) indicate conservation between groups of strongly similar properties. Dots (.) indicate conservation between groups of weakly similar properties.

## SI References


1. Y. Umena, K. Kawakami, J.-R. Shen and N. Kamiya, *Nature*, 2011, **473**, 55-60.
2. M. Zhang, M. Bommer, R. Chatterjee, R. Hussein, J. Yano, H. Dau, J. Kern, H. Dobbek and A. Zouni, *eLife*, 2017, **6**, e26933.
3. P. Jordan, P. Fromme, H. T. Witt, O. Klukas, W. Saenger and N. Krauss, *Nature*, 2001, **411**, 909-917.
4. X. Qin, M. Suga, T. Kuang and J. R. Shen, *Science*, 2015, **348**, 989-995.
5. A. Camara-Artigas, D. Brune and J. P. Allen, *Proc. Natl. Acad. Sci. USA*, 2002, **99**, 11055-11060.
6. T. Nogi, I. Fathir, M. Kobayashi, T. Nozawa and K. Miki, *Proc Natl Acad Sci U S A*, 2000, **97**, 13561-13566.
7. H. L. Axelrod, E. C. Abresch, M. Y. Okamura, A. P. Yeh, D. C. Rees and G. Feher, *J. Mol. Biol.*, 2002, **319**, 501-515.
8. J. Koepke, E. M. Krammer, A. R. Klingen, P. Sebban, G. M. Ullmann and G. Fritzsch, *J Mol Biol*, 2007, **371**, 396-409.
9. K. Saito, A. W. Rutherford and H. Ishikita, *Proc Natl Acad Sci U S A*, 2013, **110**, 7690-7695.
